# Synthesis of a *Borrelia burgdorferi*-Derived Muropeptide Standard Fragment Library

**DOI:** 10.3390/molecules29143297

**Published:** 2024-07-12

**Authors:** Rachel Putnik, Junhui Zhou, Irnov Irnov, Elise Garner, Min Liu, Klare L. Bersch, Christine Jacobs-Wagner, Catherine Leimkuhler Grimes

**Affiliations:** 1 Department of Chemistry and Biochemistry, University of Delaware, Newark, DE 19716, USA; 2 Department of Biology, Stanford University, Stanford, CA 94305, USA; 3 Sarafan Chemistry, Engineering, and Medicine for Human Health Institute, Stanford University, Stanford, CA 94305, USA; 4 Department of Microbiology and Immunology, Stanford School of Medicine, Stanford, CA 94305, USA; 5 Howard Hughes Medical Institute, Stanford University, Stanford, CA 94305, USA; 6 Department of Biological Sciences, University of Delaware, Newark, DE 19716, USA

**Keywords:** peptidoglycan (PG), Lyme disease, glycosylation, peptide coupling, innate immunity, ornithine

## Abstract

The interplay between the human innate immune system and bacterial cell wall components is pivotal in understanding diseases such as Crohn’s disease and Lyme arthritis. Lyme disease, caused by *Borrelia burgdorferi*, is the most prevalent tick-borne illness in the United States, with a substantial number of cases reported annually. While antibiotic treatments are generally effective, approximately 10% of Lyme disease cases develop persistent arthritis, suggesting a dysregulated host immune response. We have previously identified a link between the immunogenic *B. burgdorferi* peptidoglycan (PG) and Lyme arthritis and showed that this pathogen sheds significant amounts of PG fragments during growth. Here, we synthesize these PG fragments, including ornithine-containing monosaccharides and disaccharides, to mimic the unique composition of *Borrelia* cell walls, using reproducible and rigorous synthetic methods. This synthetic approach allows for the modular preparation of PG derivatives, providing a diverse library of well-defined fragments. These fragments will serve as valuable tools for investigating the role of PG-mediated innate immune response in Lyme disease and aid in the development of improved diagnostic methods and treatment strategies.

## 1. Introduction

Lyme disease, a multi-system disorder caused by the spirochete *Borrelia burgdorferi (Bb)*, is the most common tick-borne human disease in the United States [1], with nearly half a million cases diagnosed annually (https://www.cdc.gov/lyme/stats/humancases.html (accessed on 1 April 2024)). Upon infection, individuals typically exhibit erythema migrans, a distinctive “bullseye-like” rash at the site of the tick bite [2]. As the disease progresses, it can affect multiple organs at different times. Without early detection and intervention, the infection can spread to other tissues, leading to a range of health complications, including neurological disorders [2].

In the United States, the most common late-stage manifestation of Lyme disease is arthritis, characterized by inflammation, primarily in large joints, often affecting the knee. Treatment typically involves a regime of oral and intravenous antibiotics, which prove effective in most cases. However, approximately 10% of Lyme cases experience persistent arthritis months or even years following appropriate antibiotic therapy [3,4,5], resulting in conditions such as proliferative synovitis or chronic inflammatory arthritis. The persistence of Lyme disease is believed to stem from a dysregulated host immune response and the development of autoimmunity [3,6,7,8,9,10,11,12].

The peptidoglycan (PG), which is a major component of the bacterial cell wall, is known to stimulate host immune responses. We previously showed that *B. burgdorferi* PG elicits a persistent adaptive response in Lyme arthritis patients during the infectious and post-infectious phases of the disease [6]. Using an ELISA-based assay, we identified that patients with Lyme arthritis developed a specific immunoglobin G (IgG) response against *Borrelia* PG that was significantly higher in the synovial fluid samples than in the serum samples of the same patients [6]. In other words, these responses were localized to the primary site of inflammation (i.e., the joints). It was further shown that the *B. burgdorferi* PG is a persistent antigen that can remain in the synovial environment for months even after appropriate antibiotic treatment. These data strongly suggest that *Borrelia* contributes to the pathogenesis of Lyme disease.

The host immune system encounters cell wall components of *B. burgdorferi* both during transmission and after spirochetal death. *B. burgdorferi* lacks the canonical PG recycling pathways (i.e., AmpG, AmpD, LdcA) [13]. In normal bacterial cell wall biogenesis, 40–50% of PG is recycled and taken back into the cellular environment to be reincorporated into new PG [13]. However, without these enzymes, excess muropeptides accumulate in the host environment during spirochetal growth [6]. In addition, when the pathogen dies as a result of immune cell or antibiotic activities, bacterial cell lysis exposes the PG layer to the environment. Other work invokes involvement in the innate immune system, in which the host Peptidoglycan Recognition Protein 1 (PGLYRP-1), binds to PG fragments and limits their immune response [14]. While these studies undoubtedly advanced our knowledge, the discrete and complete set of fragments used in this detection scheme are not fully realized. Here, we present the synthesis of a dedicated set of PG standards derived from *Borrelia* peptidoglycan to advance the detection and treatment of this infectious bacterium.

With the increasing incidence and geographic distribution of Lyme disease cases in the US, the development of rapid and reliable methods for diagnosing this disorder is urgently needed. Current methods for assessing *Borrelia* infection are limited to serological testing. To this end, we have synthesized a suite of PG fragments containing unique modifications present in *Borrelia*. These synthetic fragments serve as invaluable tools for both the development of Lyme disease detection methods and the exploration of the immunological response of *Borrelia* PG. Future work will aim to utilize these fragments to analyze their transcriptional and immunological response in the host to better understand the etiology of Lyme disease and ultimately delineate better means of detection and therapy.

## 2. Results and Discussion

The human immune system recognizes molecular signatures known as pathogen-associated molecular patterns (PAMPs) [15,16,17]. Here, we aim to develop novel techniques for the production of bacterial cell wall-derived PAMP libraries derived from *Bb*. Our long-term goal is to leverage these molecules as tools for investigating the immune response in the context of Lyme disease.

**Figure 1:**
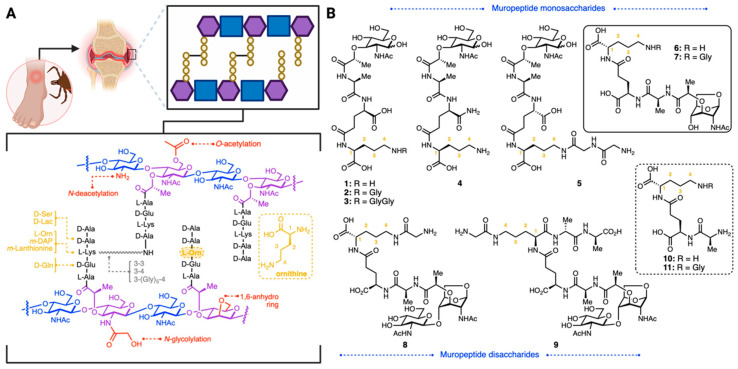
(**A**): General structure of PG cell wall and some modifications present in different bacterial species. The basic carbohydrate backbone of PG consists of GlcNAc (blue) and MurNAc (purple). *B. burgdorferi* PG features L-ornithine as the third amino acid residue (shown in yellow) and 1,6-anhydro modifications (shown in purple/red). (**B**): Synthesized borrelia-derived peptidoglycan library.

To construct the *Bb* PG library, we considered the unique structural features of the *Bb* peptidoglycan. Gram-positive bacteria typically feature l-lysine as the third residue in the peptide stem, whereas Gram-negative species often substitute lysine with *meso*-diaminopimelic acid (*m*-DAP) [18,19]. In addition, in the *Bb* PG, the second amino acid is iso-glutamate instead of iso-glutamine, which is found in the commercially available PG fragment muramyl dipeptide (MDP). This subtle switch from acid to amide can influence recognition by the immune system (Figure 1). For instance, NOD2 binds MDP, eliciting an immune response [20,21], while NOD1 detects DAP-containing fragments [22]. The peptidoglycan of *Bb* has an atypical composition: *Bb* PG replaces the canonical l-lysine or *m*-DAP with l-ornithine (Figure 1A), which may affect its immune detection [23,24]. Ornithine is further linked to glycine residues and neighboring peptidoglycan monomers to produce the rigid polymer peptidoglycan (PG).

Unmodified PG consists of alternating units of β-1,4-linked *N*-acetylglucosamine (GlcNAc) and *N*-acetylmuramic acid (MurNAc). *E. coli* and other Gram-negative bacteria terminate their glycan strands with 1,6-anhydro MurNAc residues, an intramolecular ring from C-1 to C-6 of MurNAc [25]. These 1,6-anhydromuropeptides are the product of lytic or peptidoglycan turnover by lytic transglycosylases [26,27]. The enzymatic mechanism involves the acid-mediated activation of the glycosidic bond, generating a reactive carbenium species [28]. Concomitant intramolecular trapping of MurNAc results in the formation of the 1,6-anhydro ring (Figure 1).

Anhydromuropeptides are typically generated during PG remodeling and growth. They are found in the periplasm of Gram-negative bacteria and are recycled into the cytoplasm by AmpG [29]. However, as mentioned above, *Bb* lacks the transporters to recycle these lytic fragments. These fragments have interesting applications as signaling molecules. For example, the 1,6-anhydro disaccharide tracheal cytotoxin (TCT) is a potent endotoxin [30] and has been identified in *B. pertussis* and *N. gonorrhoeae* [31,32]. TCT is cytotoxic and can elevate NOD1-mediated NF-kB expression [33]. The *Bb* variant of TCT has not been synthesized, nor have the subtle modifications at the second position (acid to amide) been produced, making it challenging to detect in patients and in subsequent downstream molecular profiling.

### Synthesis of Borrelia-Inspired Muropeptides

**Scheme 1:**
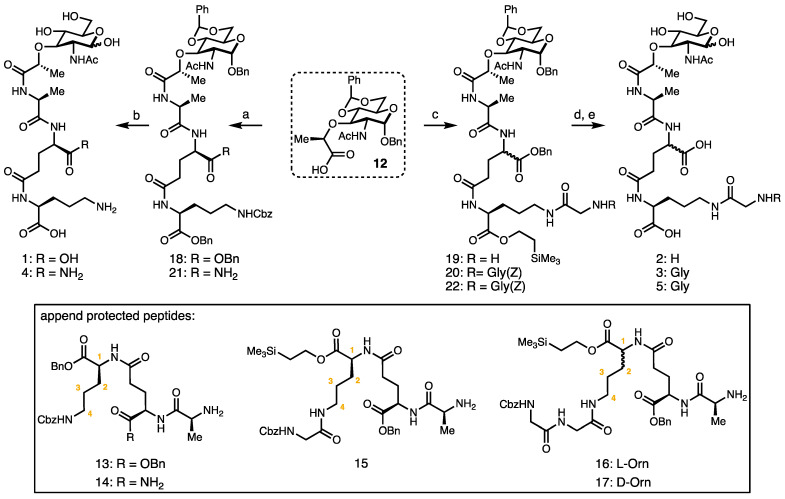
Synthetic route to l-ornithine MTP derivatives. Reagents and conditions: (a) tripeptide (**13** or **14**), HATU, DIPEA, THF, 58% or 71%, respectively; (b) H_2_, Pd(OH)_2_/C, THF/H_2_O/AcOH, 55–58%; (c) tripeptide (**15**, **16** or **17**), HATU, DIPEA, THF, 66–74%; (d) TBAF, THF, quant.; and (e) H_2_, Pd(OH)_2_/C, THF/H_2_O/AcOH, 51–58%.

Here, PG fragments with biologically relevant modifications observed in *Bb* were prepared by chemical synthesis. This is important as, had the fragments been produced from bacteria, extensive characterization would have been needed to show that potent endotoxins had not contaminated the sample. A library of fragments was carefully designed to include ornithine-containing mono- and disaccharides, shown in Figure 1B. All fragments contained the ornithine amino acid, with the corresponding amide- or carboxylic acid-terminated glutamine fragments (second position), and will be valuable resources in delineating the structural features of *Bb* that are essential for innate immune signaling and diagnostic strategies.

l-Ornithine derivatives of MTP (l-Glu-d-Orn, **1**; d-Glu-l-OrnGly, **2**; d-Glu-l-OrnGlyGly, **3**, d-isoGln-l-Orn, **4**; d-Glu-d-Orn-GlyGly, **5**; Figure 1) were synthesized from protected *N*-acetyl muramic acid (**12**, Scheme 1) following critical modifications to a literature precedent [34]. This synthesis is advantageous as it allows for the modular preparation of *Bb* PG derivatives with either the reducing ends exposed or in the 1,6-anhydro-form.

The synthesis of ornithine-containing monosaccharides commences from the common protected MurNAc intermediate; Gigg and Carroll first reported this synthesis in *Nature 1961* [35] which was subsequently modified by Melnyk et al. [34], to facilitate the rapid protection of the four and six hydroxyl groups. Here, the key chemistry developed was the coupling of discrete tripeptide units to a common core. In previous syntheses of PG fragments which utilized longer chains, the sequential addition of amino acid building blocks was required [36]. This challenging peptide coupling was realized by changing the peptide coupling reagent to HATU (hexafluorophosphate azabenzotriazole tetramethyl uranium) and increasing the ratio of peptide amine electrophile to sugar nucleophile (1.2:1). The coupling reactions of protected tripeptide fragments **13**–**17** (unprotected forms shown as **10** and **11**) to protected muramic acid **12** yielded protected MTP derivatives **18**–**22** in good yields. The hydrogenation of **18** and **21** yielded monosaccharides **1** and **4**. Global deprotection (desilylation/hydrogenation) provided ornithine PG derivatives **2**, **3,** and **5**. This synthesis provides a convenient route to muramyl tripeptide derivatives terminating with the ornithine amino acid and permitting access to the broader communities, as, currently, these compounds are not readily available to the microbiology or immunology communities.

**Scheme 2:**
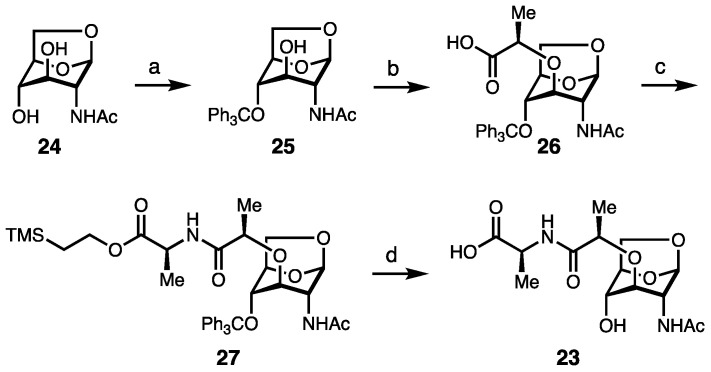
Synthesis of 1,6-anhydromuramic monopeptide **23**. Reagents and conditions: (a) Ph_3_COTf, collidine, DCM, 86%; (b) (*S*)-(−)-2-chloropropionic acid, NaH, DMF; 93%; (c) l-Ala-OTMSE, HATU, NMM, THF; 98%; and (d) TBAF, THF, quant.

1,6-Anhydro monosaccharides (**6**–**7**) and disaccharides containing ornithine fragments (**8**–**9**) were synthesized from the 1,6-anhydromuramic monopeptide (**23**, Scheme 2). The synthesis of **23** commenced from 1,6-anhydro GlcNAc **24**, which had been prepared in two steps from commercially available GlcNAc [37]. The selective protection of the 4-OH of **24** was achieved using trityl triflate, yielding **25**, which was then alkylated with (S)-(-)-2-chloropropionic acid to yield **26** [38]. The coupling of **26** and TMS-protected alanine yielded **27** [39]. Finally, the acidic removal of the trityl and silyl protecting groups afforded **23**. This differs from the reducing sugar form (**2**, **3** and **5**) synthesis, as the alanine was added first to the common core and then diversified with di- or tripeptide units to decongest the environment of amide bond formation. We attempted to add the tripeptide unit directly to the anhydro-core, and this reaction was not successful in our hands, suggesting that longer peptides can only be added after the initial alanine addition to the MurNAc 1,6-anhydro-core (Scheme 2).

**Scheme 3:**
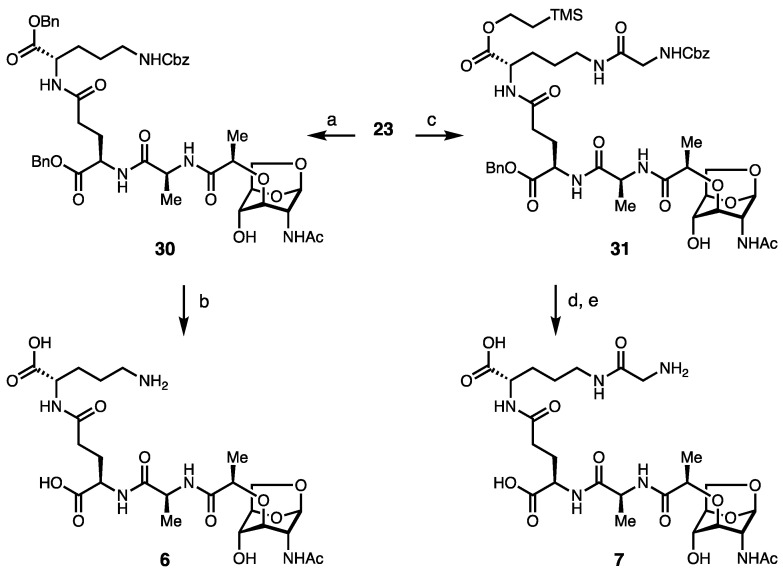
Synthesis of 1,6-anhydromuropeptides. Reagents and conditions: (a) D-Glu(OBn)-L-Orn(Z)-OBn **28**, HATU, NMM, THF, 62%; (b) H_2_, Pd(OH)_2_/C, THF, H_2_O, acetic acid, 58%; (c) D-Glu(OBn)-L-Orn(Z)-Gly **29**, HATU, NMM, THF, 56%; (d) TBAF, THF, quant; and (e) H_2_, Pd(OH)_2_/C, THF, H_2_O, acetic acid, 58%.

The coupling reactions of protected dipeptide units **28** and **29** and sugar unit **23** yielded protected 1,6-anhydromuropeptides **30** and **31** (Scheme 3). Subsequently, global deprotection was performed using Pd/C and H_2_ to provide **6** and **7**.

**Scheme 4:**
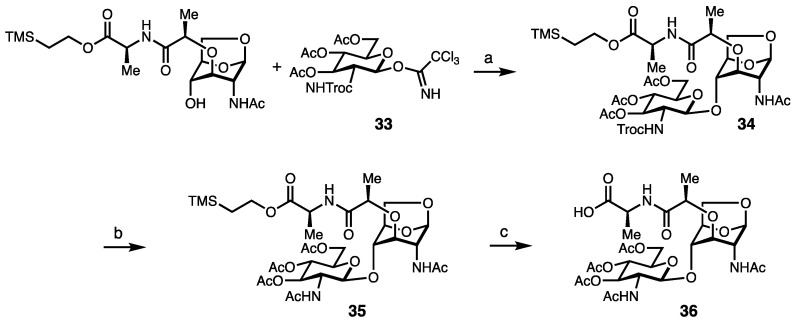
Synthesis of disaccharide intermediate **36.** Reagents and conditions: (a) TMSOTf, 4 Å molecular sieves, DCM, 70%; (b) Zn dust, THF, acetic acid, Ac_2_O; and (**c**) TBAF, THF, 83% over two steps.

1,6-Anhydro disaccharides were synthesized via the glycosylation [40] of **23** and glycosyl donor **33**, which provided the fully protected disaccharide core (Scheme 4). The trichloroacetimidate donor **34** with an *N*-((2,2,2-trichloroethyoxy)carbonyl) (*N*-Troc) used as the protecting group for the 2-NH position was optimal. It was found that 2.5 equivalents of donor **34** and 0.2 equivalents of TMSOTf under extremely anhydrous conditions gave the best consistent yields. Zn dust was used to remove the *N*-Troc protecting group from the glucosamine residue, liberating the amine, which was acetylated to yield **35**. The removal of the silyl protecting groups exposed the carboxylic acid for coupling with the remaining peptide fragments, yielding **36**.

**Scheme 5:**
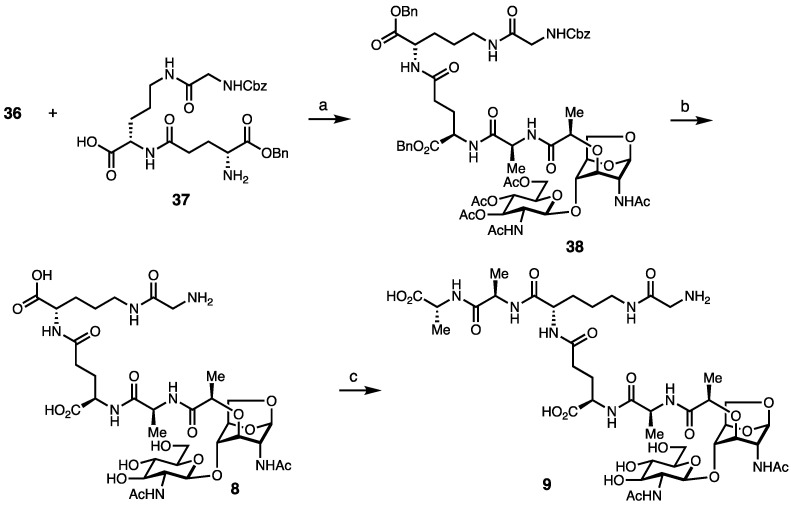
Synthesis of Borrelia disaccharides. Reagents and conditions. (a) **36**, HATU, DIPEA, THF, 73%; (b) LiOH, ACN, and then, H_2_, Pd(OH)_2_, THF, MeOH, 60% over two steps; and (c) D-Ala-D-Ala-OH, HBTU, HOBt, DMF, 55%.

Synonymous to the preparation of the anhydro-derivatives (Scheme 4), the peptide portions were synthesized separately, enabling the modular assembly of complex disaccharide muropeptides (Scheme 5). Peptide **37** was synthesized in five steps from commercially available Gly(Z), involving the consecutive coupling of l-Orn, d-*iso*-Glu, and glycine residues.

The coupling of **36** and **37** yielded **38**. Liberation of the carbohydrate hydroxyl groups, followed by hydrogenation of the benzyl-protecting groups, yielded **8**. For the synthesis of pentapeptide disaccharide **9**, a final coupling reaction used HBTU and excess d-Ala-d-Ala to **8** in a 55% yield. We note that longer peptides could not be directly added to **36,** synonymous with the peptide couplings to the anhydro-fragments, suggesting that congested environments of the sugar core limit the size of the peptide cargo to be added.

## 3. Conclusions

We have developed a streamlined protocol for producing key PG fragments from *Borrelia*, containing structural features such as ornithine, amide vs. carboxylic acid iso-glutamine, and 1,6-anhydro MurNAc backbones. The fragment library is well defined, rigorously characterized by HRMS signatures and NMRs. This robust and rigorous fragment library will play a critical role in advancing the understanding of the molecular immunology of the infectious agent *Bb*. To initially showcase utility, compounds from this library were included on a peptidoglycan fragment array and demonstrated the ability to bind the known innate immune receptor PGLYRP-1 [14,41]. Future work will include using these standards to screen innate immune responses and analyze specific PG signatures from patient-derived fluids. Ultimately the rigorous fragment library will enable the development of improved treatment regimens and detection mechanisms for this pathogen.

## 4. Experimental Section

### 4.1. Materials

All chemicals were purchased from Sigma Aldrich (St. Louis, MO, USA), ThermoFisher (Waltham, MA, USA), or ChemImpex (Wood Dale, IL, USA) and used without further purification unless otherwise noted. All solvents were reagent-grade anhydrous and purchased from Sigma Aldrich. Deuterated NMR solvents were purchased from Cambridge Isotope Laboratories (Tewksbury, MA, USA). Reactions were monitored by thin-layer chromatography (TLC) with glass plates coated with silica gel (silica HD TLC plates, UV 254, 250 μm, Sorbent Technologies (Norcross, GA, USA)) and visualized with shortwave 254 nm UV light or developed upon heating with p-Anisaldehyde or ninhydrin. Semipreparative HPLC was performed on an Agilent Series 1100 instrument (Wilmington, DE, USA) using a Phenomenex Luna 5 μm C18 100Å column (250 mm × 10 mm). Preparative HPLC purification was performed using a Waters 2767 sample manager with HPLC and SQD2 MS using a Sunfire Prep C18 OBD 5 μm 19 × 1000 mm or 4.6 × 50 mm column.

### 4.2. Instrumentation

NMR spectra were recorded on either a Bruker AVIII 400 MHz or AVIII 600 MHz spectrometer (Billerica, MA, USA). High-resolution mass spectrometry (HRMS-ESI) data were obtained at the University of Delaware Mass Spectrometry Facility (Thermo Q-Exactive Orbitrap (Waltham, MA, USA)). Low-resolution mass spectrometry (LRMS-ESI) data were obtained using an ACQUITY UPLC H-Class/SQD2 (ThermoFisher; Waltham, MA, USA).

### 4.3. General Information and Considerations

All reactions were performed in flame-dried or oven-dried flasks or vials, equipped with rubber septa, a positive pressure of nitrogen, and magnetic stirring. Unless otherwise noted, all solvents were anhydrous and transferred via a syringe.

Compound **12** was prepared according to Melnyk et al. [34]. For the full synthetic details and the characterization of compounds **1**–**38**, please see the Supplementary Materials, including Materials and Methods, Synthesis of the PG Fragment Library and all NMR Spectra.

## Data Availability

All data are available in the Supplementary Materials.

## Supplementary Materials

The following supporting information can be downloaded at https://www.mdpi.com/article/10.3390/molecules29143297/s1. References [42,43] are cited in the Supplementary Materials.
