# Supplementary material for: Synthesis of a Borrelia burgdorferi-Derived Muropeptide Standard Fragment Library
Source: Molecules. 2024 Jul 12;29(14):3297. doi: 10.3390/molecules29143297 (PMC11279244; doi:10.3390/molecules29143297)
Supplement: Supplementary file 1 [file molecules-29-03297-s001.zip › molecules-3058438-supplementary.pdf]

Supplemental Information for:

Synthesis of a *Borrelia burgdorferi*-Derived Muropeptide Standard Fragment Library

Rachel Putnik,[1] Junhui Zhou,[1] Irnov Irnov,[2] Elise Garner,[1] Min Liu,[1] Klare Lazor Bersch,[1] Christine Jacobs-Wagner,[2-5] Catherine Leimkuhler Grimes[1,6,a]

Author Information:

1. Department of Chemistry and Biochemistry, University of Delaware, Newark, DE 19716, USA
2. Department of Biology, Stanford University, Stanford, CA 94305, USA
3. Sarafan Chemistry, Engineering, and Medicine for Human Health Institute, Stanford University, Stanford, CA 94305, USA
4. Department of Microbiology and Immunology, Stanford School of Medicine, Stanford, CA 94305, USA
5. Howard Hughes Medical Institute, Stanford University, Stanford, CA 94305, USA
6. Department of Biological Sciences, University of Delaware, Newark, DE 19716, USA

The authors declare no conflict of interest.

[a]To whom correspondence should be addressed. E-mail : cgrimes@udel.edu

Table of Contents:

1. **General Materials and Methods……………………………………S2**
2. **Synthesis of the PG Fragment Library…………………………...S3**
3. **NMR Spectra…………………………………………………………S20**
4. **References…………………………………………………………...S41**

General Materials and Methods

All chemicals were purchased from Sigma Aldrich, ThermoFisher or ChemImpex and used without further purification unless otherwise noted. All solvents were reagent grade anhydrous and purchased from Sigma Aldrich. Deuterated NMR solvents were purchased from Cambridge Isotope Laboratories. Reactions were monitored by thin layer chromatography (TLC) with glass plates coated with silica gel (silica HD TLC plates, UV 254, 250 um, Sorbent Technologies) and visualized with shortwave 254 nm UV light or developed upon heating with p-Anisaldehyde or ninhydrin. Semipreparative HPLC was performed on an Agilent Series 1100 instrument using a Phenomenex Luna 5 um C18 100Å column (250 mm x 10 mm). Preparative HPLC purification was performed using a Waters 2767 sample manager with HPLC and SQD2 MS using a Sunfire Prep C18 OBD 5 um 19 x 100 mm or 4.6 x 50 mm column.

Instrumentation

NMR spectra were recorded on either a Bruker AVIII 400 MHz or AVIII 600 MHz spectrometer. High-resolution mass spectrometry (HRMS-ESI) data were obtained at the University of Delaware Mass Spectrometry Facility (Thermo Q-Exactive Orbitrap). Low-resolution mass spectrometry (LRMS-ESI) data were obtained using an ACQUITY UPLC H-Class/SQD2

General Information and Considerations

All reactions were performed in flame-dried or oven-dried flasks or vials, equipped with rubber septa, a positive pressure of nitrogen, and magnetic stirring. Unless otherwise noted, all solvents were anhydrous and transferred via a syringe.

Synthesis of the PG Fragment Library

Boc-D-Glu(OBn)-L-Orn(Z)-OBn (starting material for Boc-**13**):
Boc-D-Glu(OBn) (1.00 g, 2.96 mmol) and HBTU (1.35 g, 3.55 mmol) were dissolved in CH_2Cl_2 (50 mL), and the solution was cooled to 0 °C. Commercially available L-Orn(Z)-OBn•HCl (1.20 g, 2.96 mmol) was added, followed by NMM (850 uL, 7.70 mmol). The reaction mixture was stirred overnight at RT. The solution was extracted with DCM (x3), the combined organic phases were washed with brine, and dried. Purification by flash column chromatography on silica gel (DCM: methanol 50:1) yielded dipeptide (2.20 g) as white solid.

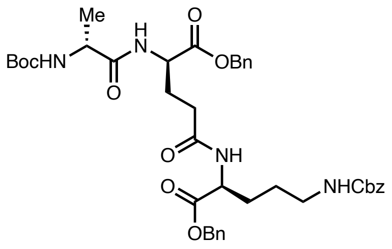

Boc-**13**

Boc-L-Ala-D-Glu(OBn)-L-Orn(Z)-OBn (Boc-**13**):
Boc-D-Glu(OBn)- L-Orn(Z)-OBn (0.500 g, 0.740 mmol) was dissolved in CH_2Cl_2 (3 mL) and 4N HCl/dioxane (1 mL) was added to the mixture. The solution was stirred for 2 h. Upon completion, the solution was poured into anhydrous ether (20 mL). After standing for 1 hour, the solution was filtered and triturated, washing with cold Et_2O (x3), to yield H-D-Glu(OBn)-L-Orn(Z)-OBn as a white solid (453 mg, quant). Subsequently, to a stirring solution of H-D-Glu(OBn)-L-Orn(Z)-OBn (453 mg, 0.740 mmol) in THF (10 mL) was added Boc-L-Ala-OSu (212 mg, 0.740 mmol). DIPEA (193 µL, 1.11 mmol) was added and the reaction was stirred overnight at RT, at which point, it was concentrated. The resulting residue was dissolved in EtOAc, washed with 0.01N HCl (x3), brine, and dried (Na_2SO_4). Precipitation by adding hexanes:EtOAc (1:1) gave the Boc-**13** (491 mg, 89 %) as a white solid. ^{1}H NMR (600 MHz, MeOD) δ 7.40 – 7.36 (m, 3H), 7.35 (d, *J* = 2.5 Hz, 3H), 7.36 – 7.29 (m, 7H), 7.32 – 7.27 (m, 1H), 5.22 (s, 1H), 5.21 – 5.12 (m, 4H), 5.06 (s, 2H), 4.48 (dd, *J* = 9.5, 4.9 Hz, 1H), 4.42 (dd, *J* = 8.9, 5.1 Hz, 1H), 4.08 (q, J = 7.0 Hz, 1H), 3.12 (t, *J* = 6.8 Hz, 2H), 2.34 – 2.27 (m, 2H), 2.19 (dd, *J* = 14.4, 8.3 Hz, 1H), 2.00 (ddd, J = 13.0, 9.1, 6.5 Hz, 1H), 1.86 (ddd, *J* = 13.6, 10.0, 6.3 Hz, 1H), 1.72 (dd, *J* = 15.7, 8.1 Hz, 1H), 1.55 (p, *J* = 7.4 Hz, 2H), 1.44 (s, 9H), 1.42 (s, 1H), 1.30 (d, *J* = 7.2 Hz, 3H), 1.27 (s, 3H). ^{13}C NMR (151 MHz, MeOD) δ 174.78, 173.50, 171.95, 171.29, 157.49, 137.03, 135.85, 135.77, 128.18, 128.06, 127.91, 127.87, 127.55, 127.36, 79.36, 66.65, 66.54, 65.96, 52.46, 51.81, 50.52, 48.03, 47.89, 47.74, 47.60, 47.46, 47.32, 47.18, 39.80, 31.36, 28.16, 27.33, 26.85, 25.92, 16.87. LRMS (ESI+) for $C_{40}H_{50}N_4O_{10}$ (746.35269): 747.310 $[M+H]^+$.

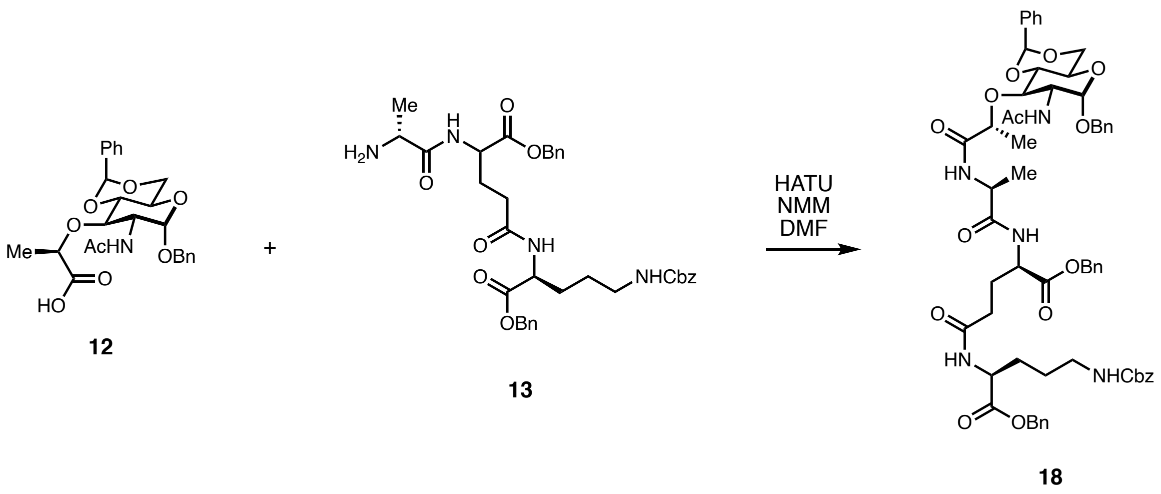

Benzyl N²-(((R)-2-(((2R,4aR,6S,7R,8R,8aS)-7-acetamido-6-(benzyloxy)-2-phenylhexahydropyrano[3,2-d][1,3]dioxin-8-yl)oxy)propanoyl)-L-alanyl)-N⁵-((S)-1-(benzyloxy)-5-(((benzyloxy)carbonyl)amino)-1-oxopentan-2-yl)-D-glutaminate (**18**): Boc-**13** (100 mg) was dissolved in DCM (1 mL) and TFA (1 mL) was added. The reaction was stirred for 2 h at RT before precipitation using diethyl ether. The resulting solid was lyophilized to give H-L-Ala-D-Glu(OBn)-L-Orn(Z)-OBn **13** (quant) as a white powder. Subsequently, to a stirring solution of **12** (334 mg, 0.707 mmol) in DMF (5 mL) was added **13** (483 mg, 0.707 mmol). *N*-Methylmorpholine (NMM) (203 µL, 1.84 mmol) and HATU (323 mg, 0.848 mmol) were added. The solution was stirred overnight and then concentrated. The residue was dissolved in EtOAc, washed with 1N HCl (x3), brine, and dried (Na_2SO_4). Purification by flash column chromatography on silica gel (1–4% MeOH in 10% ACN/CH_2Cl_2) yielded **18** as a colorless solid (550 mg, 71 %). ^{1}H NMR (600 MHz, DMSO) δ 8.46 (dd, *J* = 16.9, 7.8 Hz, 1H), 8.26 (d, *J* = 7.4 Hz, 1H), 8.15 (d, *J* = 8.3 Hz, 1H), 7.54 (d, *J* = 7.8 Hz, 1H), 7.47 – 7.42 (m, 2H), 7.42 – 7.26 (m, 18H), 7.32 (s, 8H), 7.24 (t, *J* = 5.7 Hz, 1H), 5.71 (s, 1H), 5.14 – 5.06 (m, 1H), 5.10 (s, 3H), 5.00 (s, 2H), 4.87 (d, *J* = 3.7 Hz, 1H), 4.71 (d, *J* = 12.5 Hz, 1H), 4.51 (d, *J* = 12.5 Hz, 1H), 4.35 (p, *J* = 7.1 Hz, 1H), 4.30 (td, *J* = 8.3, 7.9, 5.3 Hz, 1H), 4.27 – 4.15 (m, 3H), 4.05 – 3.98 (m, 1H), 3.79 (s, 1H), 3.76 – 3.68 (m, 1H), 3.74 (s, 2H), 3.33 (s, 16H), 2.98 (q, *J* = 6.6 Hz, 2H), 2.20 (t, *J* = 7.9 Hz, 2H), 2.01 – 1.94 (m, 1H), 1.86 – 1.79 (m, 1H), 1.80 (s, 3H), 1.74 – 1.67 (m, 1H), 1.58 (dt, *J* = 13.8, 4.8 Hz, 1H), 1.43 (dt, *J* = 9.7, 6.3 Hz, 1H), 1.27 – 1.15 (m, 6H). ^{13}C NMR (151 MHz, DMSO) δ 187.04, 172.80, 172.43, 172.16, 171.93, 170.06, 156.59, 138.06, 138.01, 137.70, 136.43, 136.28, 129.24, 128.87, 128.81, 128.72, 128.67, 128.58, 128.47, 128.22, 128.21, 128.19, 128.16, 128.09, 127.95, 127.67, 126.38, 126.33, 100.80, 97.46, 81.31, 77.38, 76.49, 69.28, 68.37, 66.48, 66.29, 65.62, 63.35, 53.63, 52.41, 52.22, 48.18, 40.53, 40.41, 40.39, 40.27, 40.25, 40.13, 39.99, 39.85, 39.71, 39.58, 31.62, 28.55, 27.37, 26.37, 23.05, 19.41. LRMS (ESI+) for $C_{60}H_{69}N_5O_{15}$ (1099.47902): 1100.648 $[M+H]^+$.

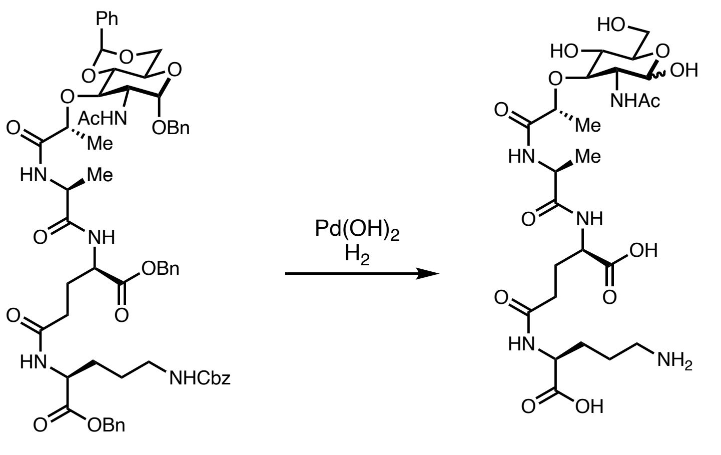

18 **1**

N^2-(((R)-2-(((3R,4R,5S,6R)-3-acetamido-2,5-dihydroxy-6- (hydroxymethyl)tetrahydro-2H-pyran-4-yl)oxy)propanoyl)-L-alanyl)-N^5-((S)-4- amino-1-carboxybutyl)-D-glutamine (**1**): **18** (550 mg, 0.500 mmol) was dissolved in THF (4 mL), acetic acid (2 mL) and water (16 mL). 20% $Pd(OH)_2$ (175 mg, 0.250 mmol) was added and the flask was evacuated three times and backfilled with hydrogen gas. After stirring for 20 hours, the reaction mixture was filtered and then condensed. The crude residue was purified using a Waters HPLC/MS with a preparative C18 column (0-45% acetonitrile in water with 0.1% formic acid over 4 minutes at 20mL/min) to yield **1** (167 mg, 55%) as white solid. ^{1}H NMR (600 MHz, D2O) δ 5.11 (d, J = 3.5 Hz, 1H), 4.62 (d, J = 8.5 Hz, 0H), 4.34 (td, J = 8.4, 5.1 Hz, 2H), 4.29 – 4.23 (m, 1H), 4.26 – 4.14 (m, 1H), 3.92 (dd, J = 10.5, 3.5 Hz, 1H), 3.88 – 3.62 (m, 3H), 3.56 – 3.39 (m, 2H), 2.98 (t, J = 7.5 Hz, 2H), 2.35 (td, J = 7.9, 7.3, 2.4 Hz, 2H), 2.22 – 2.13 (m, 1H), 2.02 – 1.95 (m, 1H), 1.97 – 1.87 (m, 4H), 1.81 – 1.65 (m, 3H), 1.38 (dd, J = 7.2, 4.0 Hz, 3H), 1.33 (dd, J = 6.7, 4.7 Hz, 3H). ^{13}C NMR (151 MHz, D2O) δ 175.74, 175.50, 175.24, 175.02, 174.83, 174.75, 174.29, 174.01, 94.93, 90.97, 82.46, 79.60, 78.04, 77.74, 75.73, 71.54, 68.95, 68.75, 60.73, 60.54, 56.16, 53.70, 52.37, 52.12, 49.59, 49.55, 38.88, 31.44, 27.56, 26.58, 23.31, 22.23, 22.01, 18.60, 16.83, 16.80. HRMS calcd for $C_{24}H_{42}N_5O_{13}^+$ (608.27009); Found 608.27828 $[M+H]^+$.

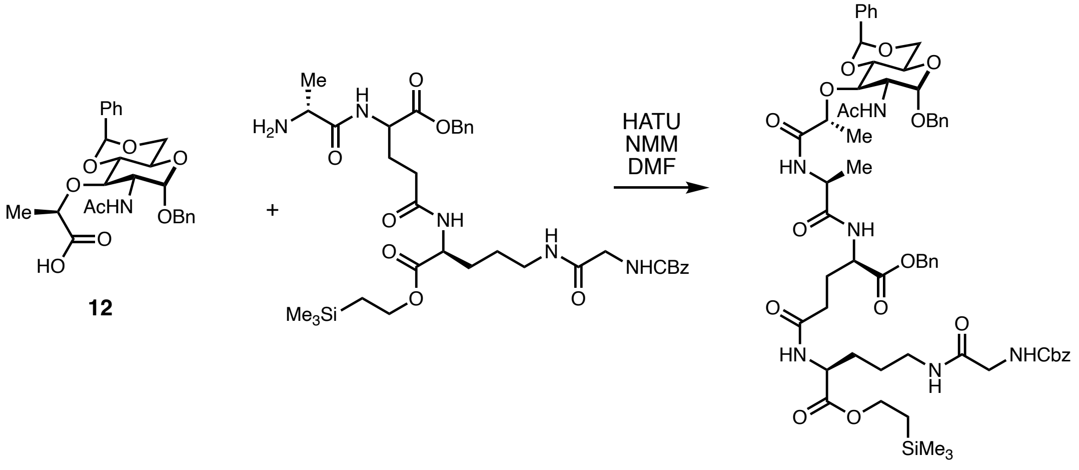

12

19

Benzyl N²-(((R)-2-(((2R,4aR,6S,7R,8R,8aS)-7-acetamido-6-(benzyloxy)-2-phenylhexahydropyrano[3,2-d][1,3]dioxin-8-yl)oxy)propanoyl)-L-alanyl)-N⁵-((S)- 5-(2-(((benzyloxy)carbonyl)amino)acetamido)-1-oxo-1-(2- (trimethylsilyl)ethoxy)pentan-2-yl)-D-glutaminate (**19**): To a solution of **12** (67 mg, 0.143 mmol) in THF (10 mL) was added H-L-Ala-D-Glu(OBn)-L-Orn(Gly(Z))-TMSE (107 mg, 0.143 mmol). NMM (41 μL, 0.372 mmol) and HATU (65 mg, 0.172 mmol) was added. The solution was stirred overnight and then concentrated. The residue was dissolved in EtOAc, and washed 0.01M HCl (x3) and brine. The organic phase was dried (Na_2SO_4), filtered, and concentrated *in vacuo*. Flash column chromatography (3–5% MeOH in 10% ACN/DCM) yielded 1**9** (110 mg, 66%) as a colorless solid. ^{1}H NMR (600 MHz, DMSO) δ 8.45 (d, *J* = 7.7 Hz, 1H), 8.18 (d, *J* = 7.5 Hz, 1H), 8.13 (d, *J* = 8.3 Hz, 1H), 7.81 (t, *J* = 5.7 Hz, 1H), 7.52 (d, *J* = 7.8 Hz, 1H), 7.44 – 7.42 (m, 1H), 7.42 – 7.16 (m, 20H, phenyl), 5.69 (s, 1H), 5.13 – 5.05 (m, 2H), 5.01 (s, 2H), 4.86 (d, *J* = 3.7 Hz, 1H), 4.69 (d, *J* = 12.5 Hz, 1H), 4.50 (d, *J* = 12.5 Hz, 1H), 4.38 – 4.25 (m, 2H), 4.22 – 4.06 (m, 5H), 3.99 (td, *J* = 9.5, 9.0, 3.7 Hz, 1H), 3.81 – 3.65 (m, 4H), 3.56 (d, *J* = 6.2 Hz, 2H), 3.02 (q, *J* = 6.5 Hz, 2H), 2.18 (t, *J* = 8.0 Hz, 2H), 1.97 (dd, *J* = 13.3, 6.3 Hz, 1H), 1.78 (s, 3H), 1.77 (s, 1H), 1.65 (s, 1H), 1.54 – 1.47 (m, 1H), 1.40 (q, *J* = 8.7 Hz, 3H), 1.21 (dd, *J* = 12.1, 6.9 Hz, 6H), 0.91 (dd, *J* = 11.3, 5.6 Hz, 2H), -0.10 (s, 9H). ^{13}C NMR (151 MHz, DMSO) δ 173.30, 173.12, 172.67, 172.48, 172.31, 170.55, 169.84, 157.42, 138.59, 138.54, 138.04, 136.82, 129.76, 129.40, 129.33, 129.25, 129.10, 128.99, 128.78, 128.72, 128.61, 126.91, 101.32, 98.00, 81.84, 77.90, 77.02, 69.81, 68.90, 66.99, 66.45, 63.87, 63.49, 54.16, 52.96, 52.76, 48.69, 44.53, 39.11, 32.15, 29.33, 27.91, 26.74, 23.58, 19.95, 19.94, 17.79, -0.50. LRMS (ESI+) for $C_{60}H_{78}N_6O_{16}Si$ (1166.52436): 1167.745 [M+H]+.

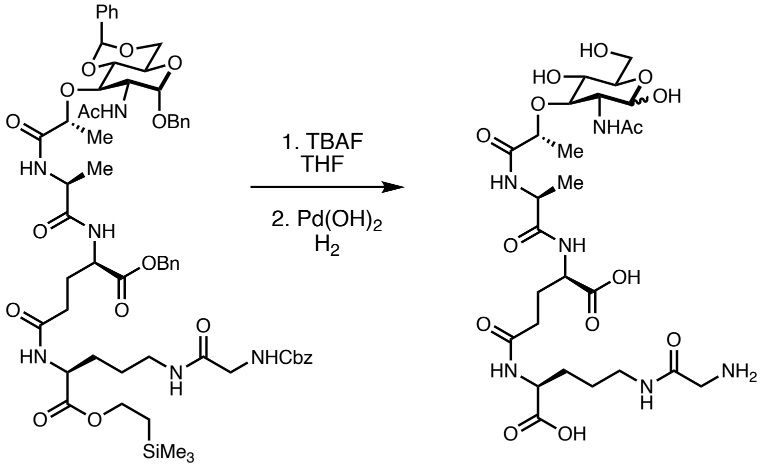

19 **2**

N²-(((R)-2-(((3R,4R,5S,6R)-3-acetamido-2,5-dihydroxy-6- (hydroxymethyl)tetrahydro-2H-pyran-4-yl)oxy)propanoyl)-L-alanyl)-N⁵-((S)-4-(2- aminoacetamido)-1-carboxybutyl)-D-glutamine (**2**) : **19** (0.075 g, 0.064 mmol) was dissolved in THF (5 mL). TBAF (1M in THF, 257 uL, 0.257 mmol) was added and the reaction was stirred overnight then condensed. The residue was dissolved in EtOAc, and washed 1N HCl (x2) and brine. The

combined organic phases were dried (Na_2SO_4), condensed, and placed under high vacuum (1 h). At which point, the crude residue was dissolved in THF (1.2 mL), acetic acid (300 uL) and water (3 mL). 20% $Pd(OH)_2$ (22 mg, 0.032 mmol) was added and the flask was evacuated three times and backfilled with hydrogen gas. After stirring for 20 h, the reaction was filtered and condensed. The crude product was purified by Waters HPLC/MS using a preparative C18 column (0-45% acetonitrile in water with 0.1% formic acid over 4 min, 20 mL/min) to yield **2** (22 mg, 51%) as white solid. ^{1}H NMR (600 MHz, D_2O) δ 5.12 (d, J = 3.5 Hz, 1H), 4.64 (d, J = 8.4 Hz, 1H), 4.31 – 4.23 (m, 2H), 4.20 (dd, J = 7.7, 5.0 Hz, 1H), 4.14 (dd, J = 8.5, 5.0 Hz, 1H), 3.89 (ddd, J = 29.6, 11.4, 2.8 Hz, 1H), 3.83 (s, 1H), 3.82 – 3.74 (m, 1H), 3.74 (s, 2H), 3.73 – 3.64 (m, 1H), 3.57 – 3.41 (m, 1H), 3.22 (t, J = 6.7 Hz, 2H), 2.26 (t, J = 7.9 Hz, 2H), 2.09 (dd, J = 14.1, 6.4 Hz, 1H), 1.95 – 1.86 (m, 4H), 1.78 (dd, J = 13.5, 5.5 Hz, 1H), 1.64 (dq, J = 14.9, 7.9 Hz, 1H), 1.51 (p, J = 7.2 Hz, 2H), 1.41 – 1.32 (m, 6H). ^{13}C NMR (151 MHz, D_2O) δ 175.71, 174.06, 166.74, 94.95, 90.95, 82.36, 79.54, 78.07, 77.79, 75.74, 71.54, 69.05, 68.86, 60.74, 60.55, 56.13, 54.51, 54.28, 53.70, 49.61, 40.43, 38.99, 32.07, 28.84, 27.91, 27.73, 24.83, 22.24, 22.01, 18.66, 16.96. HRMS *m/z*: $[M+H]^+$ calcd $C_{26}H_{45}N_6O_{14}^+$ 665.29155; Found 665.29836.

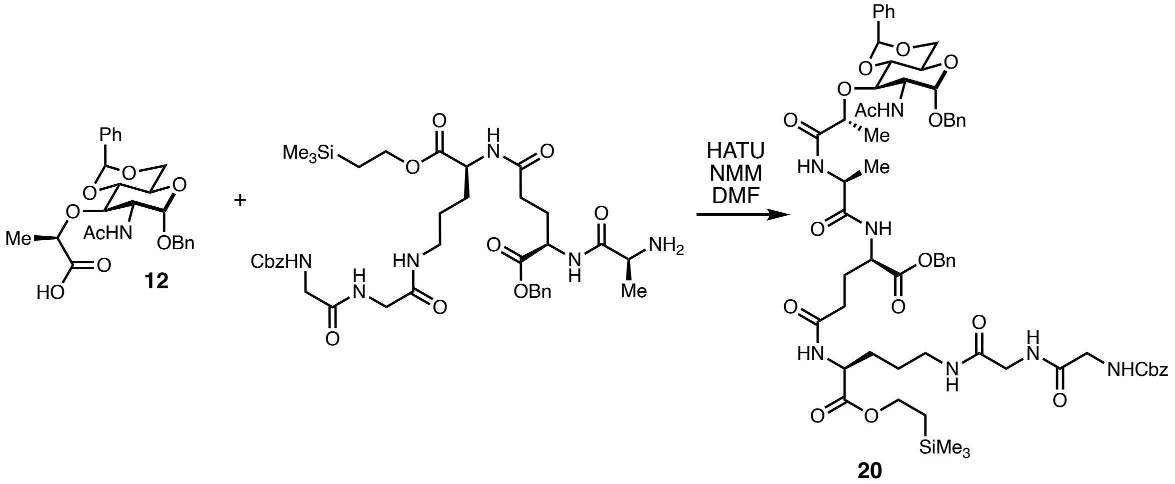

Benzyl N^2-(((R)-2-(((2R,4aR,6S,7R,8R,8aS)-7-acetamido-6-(benzyloxy)-2-phenylhexahydropyrano[3,2-d][1,3]dioxin-8-yl)oxy)propanoyl)-L-alanyl)-N^5-((S)- 5-(2-(2-(((benzyloxy)carbonyl)amino)acetamido)acetamido)-1-oxo-1-(2- (trimethylsilyl)ethoxy) pentan-2-yl)-D-glutaminate (**20**): To a stirring solution of **12** (59 mg, 0.124 mmol) in THF (10 mL) was added H-L-Ala-D-Glu(OBn)-L-Orn(GlyGly(Z))-TMSE (100 mg, 0.124 mmol). NMM (35 µL, 0.322 mmol) and HATU (57 mg, 0.149 mmol) were added. The solution was stirred overnight and then concentrated. The residue was dissolved in EtOAc, washed with 1N HCl (x3), brine, and dried (Na_2SO_4). Purification by flash column chromatography on silica gel (3–5% MeOH in 10% ACN/CH_2Cl_2) yielded **20** (90 mg, 59%) as a colorless solid. ^{1}H NMR (600 MHz, DMSO) δ 7.76 (s, 1H), 7.54 – 7.46 (m, 1H), 7.45 – 7.39 (m, 1H), 7.37 (dd, J = 15.8, 8.4 Hz, 3H), 7.35 (s, 6H), 7.33 (s, 1H), 7.34 – 7.27 (m, 1H), 7.28 (s, 1H), 5.70 (s, 1H), 5.10 (dd, J = 10.7, 6.5 Hz, 1H), 5.03 (s, 1H), 4.88 (dd, J = 18.3, 3.7 Hz, 1H), 4.69 (d, J = 12.5 Hz, 1H), 4.49 (dd, J = 17.4, 12.5 Hz, 1H), 4.37 – 4.24 (m, 1H), 4.16 (tt, J = 16.8, 7.0 Hz, 2H), 4.09 (t, J = 8.4 Hz, 1H), 4.00 (s, 1H), 3.81 – 3.74 (m, 1H), 3.74 – 3.68 (m, 2H), 3.65 (t, J = 6.2 Hz, 2H), 3.33 (s, 17H), 3.05 – 3.00 (m, 1H), 2.18 (t, J = 8.0

Hz, 1H), 2.02 – 1.94 (m, 1H), 1.80 (s, 1H), 1.79 (s, 2H), 1.65 (s, 1H), 1.41 (s, 1H), 1.22 (dt, J = 12.7, 6.9 Hz, 6H), 0.94 – 0.88 (m, 1H). ^{13}C NMR (151 MHz, DMSO) δ 173.32, 173.12, 172.70, 172.48, 172.35, 170.61, 170.38, 169.53, 157.59, 138.59, 138.54, 137.96, 136.82, 129.78, 129.41, 129.39, 129.35, 129.26, 129.23, 129.16, 129.12, 129.01, 128.82, 128.77, 128.72, 128.63, 128.58, 126.92, 126.89, 126.87, 101.34, 98.00, 81.84, 77.91, 77.03, 69.82, 68.91, 67.00, 66.57, 63.88, 63.52, 54.17, 52.97, 52.78, 48.71, 44.66, 42.99, 41.06, 40.94, 40.92, 40.80, 40.78, 40.66, 40.52, 40.38, 40.24, 40.10, 39.14, 32.16, 30.01, 29.33, 27.91, 26.70, 23.58, 20.07, 19.94, 19.40, 17.79, -0.50. LRMS (ESI+) for $C_{62}H_{81}N_7O_{17}Si$ (1223.546): 1224.618 $[M+H]^+$.

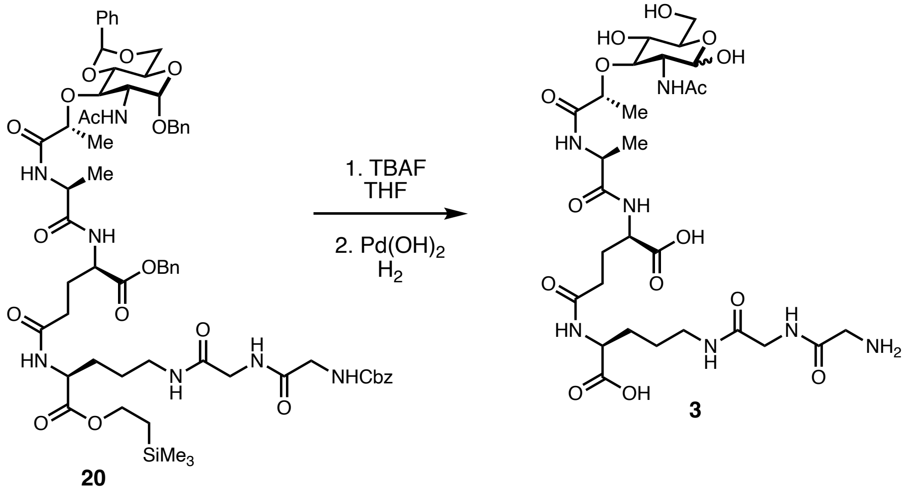

20 **3**

N[2]*-(((R)-2-(((3R,4R,5S,6R)-3-acetamido-2,5-dihydroxy-6-(hydroxymethyl)tetrahydro-2H-pyran-4-yl)oxy)propanoyl)-L-alanyl)-N*[5]*-((S)-4-(2-(2-aminoacetamido)acetamido)-1-carboxybutyl)-D-glutamine*(**3**): **20** (50 mg, 0.041 mmol) was dissolved in THF (5 mL). TBAF (1M in THF, 163 uL, 0.163 mmol) was added and the solution was stirred until complete, and then condensed. The residue was dissolved in EtOAc, washed with 1N HCl, dried (Na_2SO_4), concentrated, a placed under high vacuum for 1 h. Subsequently, the crude product was dissolved in THF (1.2 mL), acetic acid (300 uL) and water (3.6 mL). 20% $Pd(OH)_2$ (14 mg, 0.020 mmol) was added, and the flask was evacuated three times and backfilled with hydrogen. After 20 hours, the reaction mixture was filtered and condensed. The crude residue was purified by Waters HPLC/MS using a preparative C18 column (0- 45% acetonitrile in water with 0.1% formic acid over 4 min, 20 mL/min) to yield as white solid (15 mg, 0.021 mmol, 52%).^{1}H NMR (600 MHz, D_2O) δ 5.11 (d, J = 3.5 Hz, 1H), 4.32 – 4.13 (m, 4H), 3.90 (s, 2H), 3.84 (s, 2H), 3.84 – 3.60 (m, 3H), 3.57 – 3.39 (m, 2H), 3.18 (t, J = 6.7 Hz, 2H), 2.27 (t, J = 7.8 Hz, 2H), 2.09 (ddd, J = 13.6, 11.1, 6.4 Hz, 1H), 1.99 – 1.87 (m, 4H), 1.76 (ddd, J = 13.6, 9.6, 5.5 Hz, 1H), 1.62 (dq, J = 14.5, 8.1 Hz, 1H), 1.50 (p, J = 7.1 Hz, 2H), 1.35 (ddd, J = 24.3, 7.0, 4.4 Hz, 6H). ^{13}C NMR (151 MHz, D_2O) δ 177.84, 176.89, 175.71, 175.45, 174.93, 174.28, 174.21, 174.01, 170.95, 167.74, 94.95, 90.96, 82.37, 79.55, 78.06, 77.79, 75.74, 71.55, 69.05, 68.86, 60.75, 60.56, 56.14, 54.13, 53.89, 53.70, 49.61, 49.57, 49.39, 47.57, 42.52, 40.48, 38.88, 31.95, 28.62, 28.31, 27.68, 27.65, 24.84, 22.25, 22.02, 18.66, 18.64, 16.95, 16.93. HRMS *m/z:* $[M+H]^+$ calcd $C_{28}H_{48}N_7O_{15}^+$ 722.31301; Found 722.31799.

Boc-L-Ala-D-isoGln-L-Orn(Z)-OBn (Boc-**14**): Commercially available Boc-L-Ala-OSu (1.0 g, 2.96 mmol) and D-isoGln(OBn)-NH_2•HCl (0.86 g, 3.5 mmol) were dissolved in dry THF (70 mL). DIPEA (1.44 mL, 4.44 mmol) was added and the reaction mixture was stirred overnight at RT. The reaction was quenched with saturated aqueous $NaHCO_3$ solution. The solution was extracted with EtOAc (x3), dried (Na_2SO_4) and condensed. The crude product was recrystallized from EtOAc/hexanes to yield Boc-**14** (1.2 g) as a white powder. ^{1}H NMR (400 MHz, MeOD) δ 7.41 – 7.27 (m, 9H), 5.23 – 5.12 (m, 2H), 5.07 (s, 2H), 4.44 (dd, *J* = 8.9, 5.1 Hz, 1H), 4.35 (dd, *J* = 9.6, 4.4 Hz, 1H), 4.04 (q, *J* = 7.1 Hz, 1H), 3.26 (s, 1H), 3.13 (t, *J* = 6.7 Hz, 2H), 2.34 (t, *J* = 8.0 Hz, 2H), 2.19 (s, 1H), 2.01 – 1.81 (m, 2H), 1.79 – 1.65 (m, 1H), 1.55 (p, *J* = 7.2 Hz, 2H), 1.45 (s, 9H), 1.32 (d, *J* = 7.0 Hz, 3H). ^{13}C NMR (101 MHz, MeOD) δ 174.91, 173.79, 171.98, 157.50, 156.60, 137.02, 135.84, 128.20, 128.06, 127.93, 127.89, 127.56, 127.37, 79.36, 66.53, 65.95, 52.49, 52.41, 50.65, 48.24, 48.02, 47.81, 47.60, 47.38, 47.17, 46.96, 39.77, 31.58, 28.17, 27.47, 27.31, 25.93, 16.27. LRMS (ESI+) for $C_{33}H_{45}N_5O_9$ (655.749): 656.381 $[M+H]^+$.

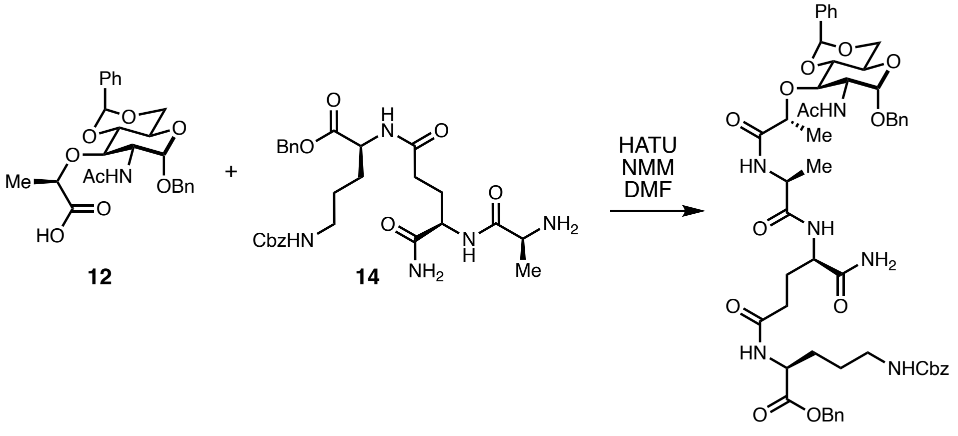

Benzyl (S)-2-((R)-4-((S)-2-((R)-2-(((2R,4aR,6S,7R,8R,8aS)-7-acetamido-6- (benzyloxy)-2-phenylhexahydropyrano[3,2-d][1,3]dioxin-8-yl)oxy)propanamido)propanamido)-5-amino-5-oxopentanamido)-5- (((benzyloxy)carbonyl)amino)pentanoate (**21**): Boc-**14** (1.2 g) was dissolved in DCM (5 mL) and TFA (5 mL) was added. The reaction was stirred for 5 h at RT before precipitation using diethyl ether. The resulting precipitate was collected by filtration and lyophilized to give TFA•NH_2-L-Ala-D-isoGln-L-Orn(Z)-OBn **14** (quant) as a white powder. Subsequently, to a stirring solution of **12** (399 mg, 0.846 mmol) in DMF (5 mL) was added **14** (500 mg, 0.846 mmol). NMM (240 µL, 1.840 mmol) and HATU (386 mg, 1.015 mmol) were added. The solution was stirred overnight at RT and then concentrated. The crude residue was dissolved in EtOAc, washed with 1N HCl (x3), brine, and dried (Na_2SO_4). Purification by flash column chromatography on silica gel (3–5% MeOH in 10% ACN/DCM) yielded **21** (495 mg, 58%) as a colorless solid. ^{1}H NMR (600 MHz, DMSO) δ 8.27 (d, *J* = 7.5 Hz, 1H), 8.17 – 8.08 (m, 2H), 7.55 (d, *J* = 7.0 Hz, 1H), 7.47 – 7.40 (m, 2H), 7.40 (s, 1H), 7.39 – 7.28 (m, 19H), 7.23 (d, *J* = 5.9 Hz, 1H), 7.06 (s, 1H), 5.72 (d, *J* = 4.4 Hz, 1H), 5.11 (s, 2H), 5.00 (s, 2H), 4.95 (s, 1H), 4.87 (d, *J* = 3.7 Hz, 1H), 4.72 (d, *J* = 12.5 Hz, 1H), 4.63 – 4.50 (m, 1H), 4.32 – 4.27 (m, 1H), 4.24 (d, *J* = 7.0 Hz, 1H), 4.22 – 4.13 (m, 3H), 4.03 (t, *J* = 7.4 Hz, 1H), 3.82 – 3.66 (m, 3H), 2.98 (q, *J* =

6.3 Hz, 2H), 2.17 – 2.11 (m, 2H), 1.93 (s, 1H), 1.82 (d, *J* = 5.5 Hz, 3H), 1.76 – 1.67 (m, 1H), 1.71 (s, 2H), 1.61 – 1.56 (m, 1H), 1.43 (q, *J* = 14.1, 10.4 Hz, 2H), 1.22 (tt, *J* = 13.1, 7.3 Hz, 7H). ^{13}C NMR (151 MHz, DMSO) δ 173.56, 172.49, 172.35, 170.15, 156.59, 138.06, 138.01, 137.71, 136.45, 129.24, 128.89, 128.82, 128.74, 128.68, 128.59, 128.46, 128.22, 128.18, 128.15, 128.10, 127.95, 127.69, 126.38, 126.33, 101.63, 100.79, 97.49, 81.29, 77.38, 76.57, 70.56, 69.26, 68.37, 66.27, 66.20, 65.62, 63.36, 53.59, 52.56, 52.41, 48.59, 40.52, 40.41, 40.39, 40.27, 40.25, 40.13, 39.99, 39.85, 39.71, 39.57, 31.99, 28.52, 28.38, 26.41, 23.08, 19.43, 19.38, 18.93, 18.79, 14.56. LRMS (ESI+) for $C_{53}H_{64}N_6O_{14}$ (1008.44805): 1009.548 $[M+H]^+$.

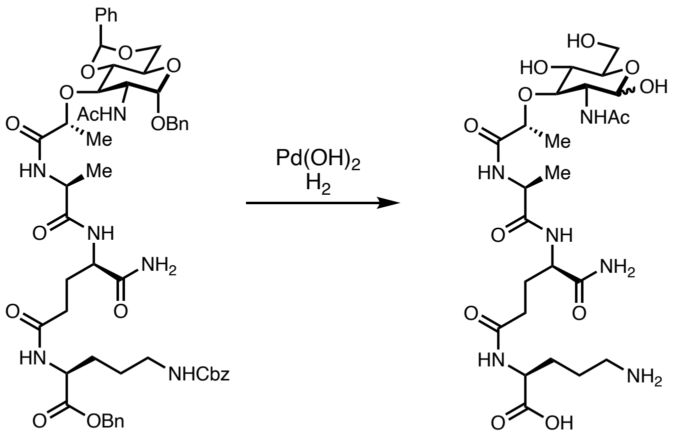

21 **4**

(S)-2-((R)-4-((S)-2-((R)-2-(((3R,4R,5S,6R)-3-acetamido-2,5-dihydroxy-6-(hydroxymethyl)tetrahydro-2H-pyran-4-yl)oxy)propanamido)propanamido)-5-amino-5-oxopentanamido)-5-aminopentanoic acid (**4**): **21** (50 mg, 0.050 mmol) was dissolved in THF (0.4 mL), acetic acid (0.2 mL) and water (1.6 mL). 20% $Pd(OH)_2$ (17.5 mg, 0.025 mmol) was added and the flask was evacuated three times and backfilled with hydrogen gas. After stirring for 20 h, the reaction mixture was filtered and condensed. The crude residue was purified by Waters HPLC/MS using a preparative C18 column (0-45% acetonitrile in water with 0.1% formic acid over 4 mins, 20 mL/min) to yield **4** (17 mg, 58%) as white solid. ^{1}H NMR (600 MHz, D_2O) δ 5.11 (t, J = 2.7 Hz, 1H), 4.63 (dd, J = 8.5, 1.9 Hz, 1H), 4.30 – 4.21 (m, 3H), 4.17 (td, J = 6.2, 5.5, 3.2 Hz, 1H), 4.06 – 4.02 (m, 1H), 3.89 – 3.75 (m, 1H), 3.78 – 3.63 (m, 1H), 3.56 – 3.40 (m, 1H), 2.97 (t, J = 7.4 Hz, 2H), 2.36 (t, J = 7.5 Hz, 2H), 2.19 – 2.09 (m, 1H), 1.94 (s, 2H), 2.01 – 1.91 (m, 3H), 1.82 (t, J = 7.7 Hz, 1H), 1.68 (dp, J = 23.4, 7.4 Hz, 4H), 1.38 (dt, J = 6.4, 2.9 Hz, 3H), 1.33 (ddd, J = 6.9, 4.9, 1.9 Hz, 3H). ^{13}C NMR (151 MHz, D_2O) δ 177.84, 175.94, 175.93, 175.71, 175.11, 174.46, 174.27, 174.00, 94.93, 90.96, 82.54, 79.64, 78.05, 77.73, 75.73, 71.53, 68.96, 68.74, 67.56, 60.73, 60.54, 56.15, 54.29, 53.68, 53.04, 52.97, 49.74, 49.72, 39.03, 31.81, 28.43, 26.93, 26.91, 23.38, 22.23, 22.01, 18.66, 18.65, 16.59, 16.54. HRMS *m/z:* $[M+H]^+$ calcd $C_{24}H_{43}N_6O_{12}^+$ 607.28607; Found 607.29382.

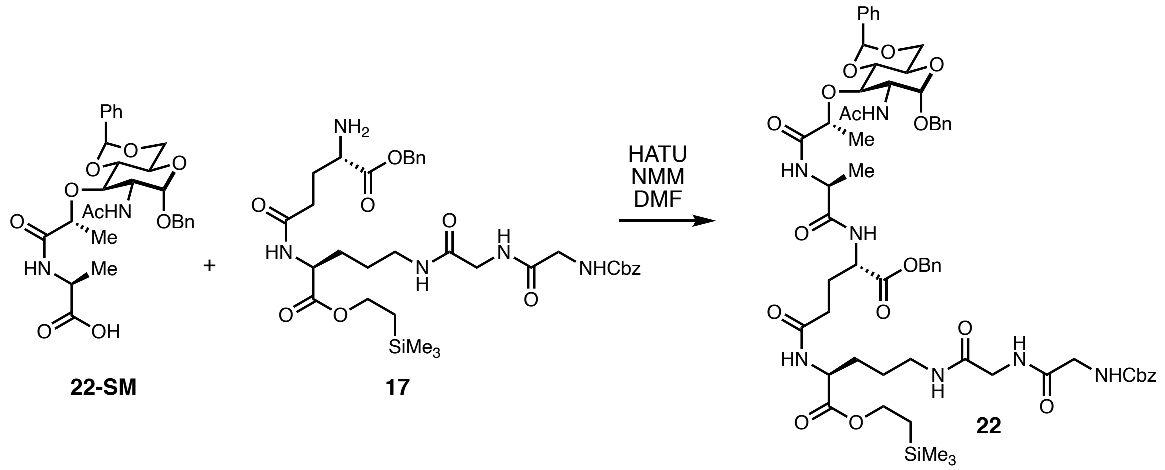

Benzyl N^2-(((R)-2-(((2R,4aR,6S,7R,8R,8aS)-7-acetamido-6-(benzyloxy)-2-phenylhexahydropyrano[3,2-d][1,3]dioxin-8-yl)oxy)propanoyl)-L-alanyl)-N^5-((R)- 5-(2-(2-(((benzyloxy)carbonyl)amino)acetamido)acetamido)-1-oxo-1-(2 (trimethylsilyl)ethoxy) pentan-2-yl)-D-glutaminate (**22**): **22-SM**[1] (158 mg, 0.292 mmol) and **17** (306 mg, 0.438 mmol) was dissolved in THF (10 mL) and the solution was cooled to 0 °C. NMM (0.1 mL, 0.876 mmol) and HATU (0.166 g, 0.438 mmol) were added. The solution was warmed to RT, stirred overnight, and condensed. The residue was dissolved in EtOAc, washed with 1N HCl (x3), brine, and dried (Na_2SO_4). Flash column chromatography on silica gel (3–5% MeOH in 10% ACN/DCM) yielded **22** (263 mg, 74%) as a colorless solid. ^{1}H NMR (600 MHz, DMSO) δ 8.47 (d, *J* = 7.9 Hz, 1H), 8.16 (d, *J* = 7.3 Hz, 1H), 8.14 (d, *J* = 8.4 Hz, 1H), 8.07 (d, *J* = 6.0 Hz, 1H), 7.75 (t, *J* = 5.8 Hz, 1H), 7.54 – 7.46 (m, 2H), 7.43 (d, *J* = 7.1 Hz, 2H), 7.36 (s, 7H), 7.41 – 7.30 (m, 7H), 7.30 – 7.25 (m, 3H), 5.70 (s, 1H), 5.13 – 5.05 (m, 2H), 5.03 (s, 2H), 4.86 (d, *J* = 3.7 Hz, 1H), 4.69 (d, J = 12.5 Hz, 1H), 4.50 (d, J = 12.5 Hz, 1H), 4.33 (p, J = 6.9 Hz, 1H), 4.30 – 4.25 (m, 1H), 4.22 – 4.13 (m, 3H), 4.08 (t, *J* = 8.4 Hz, 2H), 4.01 (dq, *J* = 11.8, 7.0 Hz, 1H), 4.00 (s, 1H), 3.77 (d, *J* = 9.0 Hz, 1H), 3.72 (s, 3H), 3.65 (d, *J* = 7.5 Hz, 4H), 3.32 (s, 15H), 3.03 (q, *J* = 6.6 Hz, 2H), 2.18 (t, *J* = 7.9 Hz, 2H), 1.98 (d, *J* = 4.6 Hz, 2H), 1.79 (s, 3H), 1.76 (s, 1H), 1.65 (s, 1H), 1.53 (d, *J* = 14.5 Hz, 1H), 1.41 (s, 2H), 1.24 – 1.14 (m, 6H), 1.07 (s, 1H), 0.91 (t, *J* = 8.4 Hz, 2H), -0.00 (s, 7H). ^{13}C NMR (151 MHz, DMSO) δ 173.31, 173.13, 172.72, 172.49, 172.28, 170.60, 170.36, 169.51, 157.58, 138.59, 138.54, 137.96, 136.82, 129.77, 129.41, 129.35, 129.26, 129.21, 129.11, 129.00, 128.81, 128.72, 128.63, 126.91, 101.32, 98.00, 81.82, 77.91, 77.04, 69.82, 68.90, 66.99, 66.56, 63.88, 63.49, 60.76, 54.16, 52.92, 52.62, 48.74, 44.64, 42.98, 41.06, 40.94, 40.92, 40.80, 40.78, 40.66, 40.53, 40.39, 40.25, 40.11, 39.13, 32.06, 29.35, 27.78, 26.66, 23.58, 21.77, 19.93, 17.79, 15.09, -0.50. LRMS (ESI+) for $C_{62}H_{81}N_7O_{17}Si$ (1223.546): 1224.618 $[M+H]^+$.

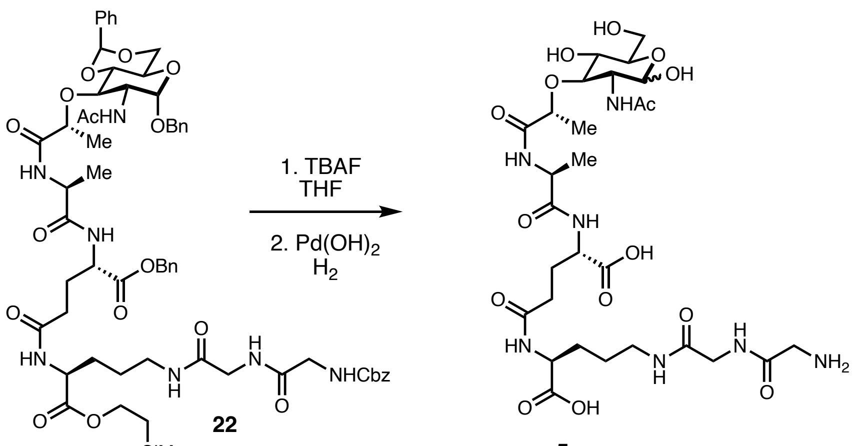

22 → **5**

N^2-(((R)-2-(((3R,4R,5S,6R)-3-acetamido-2,5-dihydroxy-6- (hydroxymethyl)tetrahydro-2H-pyran-4-yl)oxy)propanoyl)-L-alanyl)-N^5-((R)-4-(2-(2-aminoacetamido)acetamido)-1-carboxybutyl)-D-glutamine (**5**): **22** (120 mg, 0.098 mmol) was dissolved in THF (4.8 mL) and cooled to 0 °C. TBAF (1M in THF, 392 uL, 0.392 mmol) was added. The solution was stirred overnight, and condensed. The residue was dissolved in EtOAc, washed with 1N HCl, dried, and condensed. The residue was dissolved in THF (4.8 mL), acetic acid (2.4 mL) and water (16.8 mL). 20% $Pd(OH)_2$ (34 mg, 0.049 mmol) was added and the flask was evacuated three times and backfilled with hydrogen gas. After 20 h, the reaction was filtered and condensed. The crude product was purified by Waters HPLC/MS using a preparative C18 column (0-45% acetonitrile in water with 0.1% formic acid over 4 mins, 20 mL/min) to yield **5** as a white solid (39.8 mg, 52%). ^{1}H NMR (600 MHz, D2O) δ 4.72 (s, 20H), 4.32 (ddd, *J* = 14.4, 9.3, 5.0 Hz, 2H), 4.28 – 4.21 (m, 2H), 4.18 (q, J = 6.7 Hz, 0H), 3.91 (s, 2H), 3.84 (s, 2H), 3.89 – 3.77 (m, 2H), 3.77 – 3.63 (m, 2H), 3.56 – 3.39 (m, 2H), 3.20 (t, J = 6.8 Hz, 2H), 2.35 (s, 1H), 2.36 – 2.29 (m, 1H), 2.20 (dt, J = 13.8, 6.5 Hz, 1H), 1.99 – 1.91 (m, 4H), 1.83 (dq, J = 14.5, 8.2, 7.2 Hz, 1H), 1.72 – 1.63 (m, 1H), 1.59 – 1.49 (m, 2H), 1.39 (dd, J = 7.3, 4.2 Hz, 3H), 1.33 (dd, J = 6.8, 4.7 Hz, 3H). 13C NMR (151 MHz, D2O) δ 179.12, 175.97, 175.90, 175.68, 175.11, 174.27, 174.01, 170.88, 167.73, 94.94, 90.96, 82.51, 79.60, 78.04, 77.73, 75.74, 71.55, 69.01, 68.78, 60.74, 60.55, 58.65, 56.15, 55.20, 53.69, 53.07, 49.75, 49.73, 42.53, 40.45, 39.13, 33.77, 31.92, 31.14, 27.87, 26.95, 22.43, 22.24, 22.02, 18.65, 16.60. HRMS *m/z*: [M+H]+ Calcd. for $C_{28}H_{48}N_7O_{15}^+$ 722.31301; Found 722.31803.

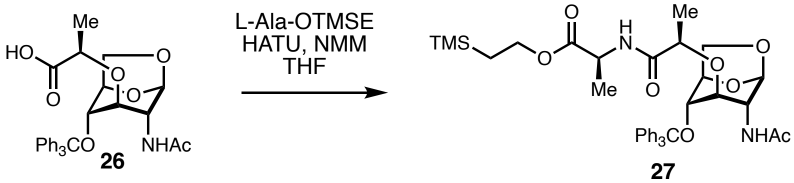

26 → **27**

2-(Trimethylsilyl)ethyl ((R)-2-(((1R,2S,3R,4R,5R)-4-acetamido- 2-(trityloxy)-6,8-dioxabicyclo[3.2.1] octan-3-yl)oxy)propanoyl)-L-alaninate (**27**): To a solution of **26** (517 mg, 1.000 mmol) in THF (20 mL) was added H-L-Ala-OTMSE[2] (284 mg, 1.5 mmol). NMM (330 µL, 3.0 mmol) and HATU (570 mg, 1.5 mmol) were added. The reaction was stirred overnight at RT, and then condensed. The crude residue was dissolved in EtOAc, and

washed with 0.01N HCl (x3) and brine, dried (Na_2SO_4), filtered, and concentrated *in vacuo*. Flash column chromatography (0–1% MeOH in 5% ACN/DCM) yielded **27** (675 mg, 0.98 mmol, 98%) as a colorless solid. 1H NMR (600 MHz, $CDCl_3$) δ 7.82 (d, *J* = 8.1 Hz, 1H), 7.50 – 7.45 (m, 6H), 7.36 – 7.28 (m, 6H), 7.31 – 7.23 (m, 3H), 6.32 (d, *J* = 9.5 Hz, 1H), 5.37 (t, *J* = 2.1 Hz, 1H), 4.54 – 4.45 (m, 1H), 4.18 – 4.09 (m, 3H), 4.00 – 3.95 (m, 1H), 3.73 (q, *J* = 6.6 Hz, 2H), 3.69 (d, *J* = 2.0 Hz, 1H), 3.52 (dd, *J* = 7.6, 5.8 Hz, 1H), 3.11 (p, *J* = 1.6 Hz, 1H), 2.01 (s, 3H), 1.37 (dd, *J* = 15.2, 7.2 Hz, 3H), 1.15 (d, *J* = 6.7 Hz, 3H), 1.03 – 0.92 (m, 2H), 0 (m, 9H). ^{13}C NMR (151 MHz, $CDCl_3$) δ 174.66, 173.52, 170.74, 145.27, 130.23, 130.08, 129.81, 129.33, 128.99, 101.86, 90.29, 79.62, 77.55, 76.78, 73.26, 66.69, 65.47, 65.00, 49.74, 49.16, 48.65, 24.81, 20.18, 19.62, 19.29, 18.85, 18.79, 0.00. LRMS (ESI+) for $C_{38}H_{48}N_2O_8Si$ (688.32): 711.79 $[M+Na]^+$.

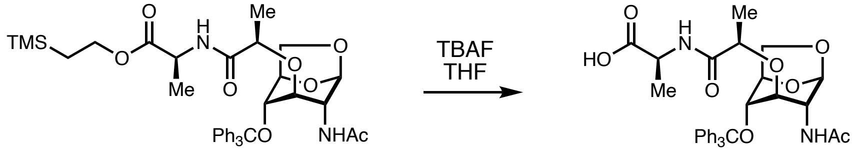

27 **23**

N^2-(((R)-2-(((1R,2S,3R,4R,5R)-4-acetamido-2-hydroxy-6,8- dioxabicyclo[3.2.1]octan-3-yl)oxy)propanoyl)- L-alanyl)-N^5-((S)-4-(2- aminoacetamido)-1-carboxybutyl)- D-glutamine (**6**): Prepared following previous literature[3] precedent. ^1H NMR (600 MHz, D_2O) δ 8.41 (s, 1H), 5.44 (d, *J* = 1.7 Hz, 1H), 4.64 (s, 1H), 4.36 (q, J = 7.1 Hz, 1H), 4.26 – 4.21 (m, 1H), 4.15 (dd, J = 7.4, 4.5 Hz, 2H), 4.10 (dd, *J* = 8.3, 4.8 Hz, 1H), 3.88 (d, *J* = 2.0 Hz, 1H), 3.84 (d, *J* = 1.9 Hz, 1H), 3.77 (dd, *J* = 7.8, 5.8 Hz), 3.73 (s, 2H), 3.39 (p, *J* = 1.6 Hz, 1H), 3.21 (t, *J* = 6.7 Hz, 2H), 2.27 – 2.20 (m, 2H), 2.07 (dt, *J* = 14.8, 7.5 Hz, 1H), 1.96 (s, 3H), 1.90 (dt, *J* = 14.9, 7.4 Hz, 1H), 1.75 (s, 1H), 1.62 (dq, *J* = 15.0, 7.8 Hz, 1H), 1.50 (h, *J* = 7.5 Hz, 2H), 1.39 (d, *J* = 7.2 Hz, 3H), 1.34 (d, *J* = 6.8 Hz, 3H). ^{13}C NMR (151 MHz, D_2O) δ 178.86, 175.49, 174.86, 173.88, 173.62, 166.79, 99.97, 78.43, 75.92, 68.23, 65.22, 54.83, 54.58, 49.46, 40.46, 39.04, 32.17, 29.03, 28.07, 24.85, 21.83, 18.02, 17.16. HRMS-ESI. Calculated $C_{26}H_{43}N_6O_{13}$ $[M+H]^+$ 647.28881; observed, 647.28814.

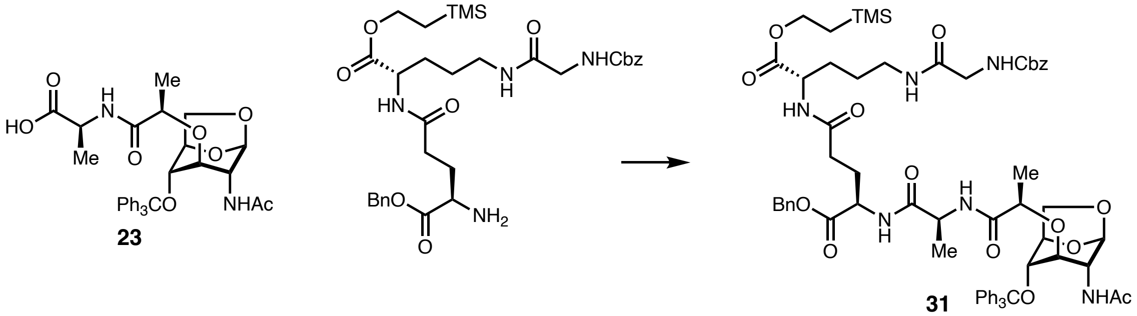

23 **31**

Benzyl N^2-(((R)-2-(((1R,2S,3R,4R,5R)-4-acetamido-2-(trityloxy)-6,8-dioxabicyclo[3.2.1]octan-3-yl)oxy)propanoyl)-L-alanyl)-N^5-((S)-5-(2- (((benzyloxy) carbonyl)amino)acetamido)-1-oxo-1-(2- (trimethylsilyl)ethoxy)pentan-2-yl)-D-glutaminate (**31**): To a stirring solution of **23** (100 mg, 0.17 mmol) in THF (5 mL) was added H-L-Ala-D-Glu(OBn)-L-Orn(TMSE)-Gly(Z) (164 mg, 0.255 mmol). NMM (56 uL, 0.51 mmol) and HATU (97 mg, 0.255 mmol). The reaction was stirred overnight, and condensed. The

residue was dissolved in EtOAc, and washed with 0.01N HCl and brine, dried over Na_2SO_4, filtered, and concentrated *in vacuo*. Flash column chromatography (2–5% MeOH in 5% ACN/DCM) yielded **31** (116 mg, 56%) as a colorless solid. ^{1}H NMR (400 MHz, $CDCl_3$) δ 7.93 (d, *J* = 5.4 Hz, 1H), 7.57 – 7.42 (m, 6H), 7.32 (dq, *J* = 11.3, 6.7 Hz, 19H), 7.05 (t, *J* = 5.1 Hz, 1H), 6.40 (d, *J* = 9.3 Hz, 1H), 6.16 (d, *J* = 5.9 Hz, 1H), 5.36 (s, 1H), 5.15 – 5.04 (m, 4H), 4.42 (qd, *J* = 8.9, 4.1 Hz, 2H), 4.13 (ddd, *J* = 32.2, 14.2, 7.2 Hz, 4H), 3.93 (dt, *J* = 18.8, 9.3 Hz, 1H), 3.87 – 3.77 (m, 1H), 3.71 (d, *J* = 5.5 Hz, 2H), 3.52 (t, *J* = 6.6 Hz, 1H), 3.40 – 3.30 (m, 1H), 3.18 – 3.08 (m, 1H), 3.02 (s, 1H), 2.92 (s, 1H), 2.58–2.35 (m, 1H), 2.27–2.15 (m, 1H), 2.10–1.92 (m, 1H), 2.01 (s, 4H), 1.86 (d, *J* = 13.3 Hz, 1H), 1.68 (m, 1H), 1.54 (d, *J* = 7.2 Hz, 2H), 1.31 (d, *J* = 7.1 Hz, 3H), 1.10 (d, *J* = 6.8 Hz, 3H), 0.99 (dt, *J* = 13.0, 4.3 Hz, 2H), 0.03 (s, 9H). ^{13}C NMR (101 MHz, $CDCl_3$) δ 173.78, 173.59, 172.82, 172.17, 171.19, 169.54, 169.50, 165.71, 156.79, 143.68, 136.39, 135.61, 128.60, 128.57, 128.48, 128.30, 128.22, 128.07, 127.81, 100.18, 88.68, 78.42, 77.49, 77.17, 66.88, 66.64, 65.08, 63.77, 47.14, 45.42, 44.46, 38.62, 30.63, 28.54, 26.47, 25.26, 23.27, 17.97, 17.31, 17.02, -1.49. LRMS (ESI+) for $C_{65}H_{80}N_6O_{15}Si$ (1212.545): 1235.392 $[M+Na]^+$.

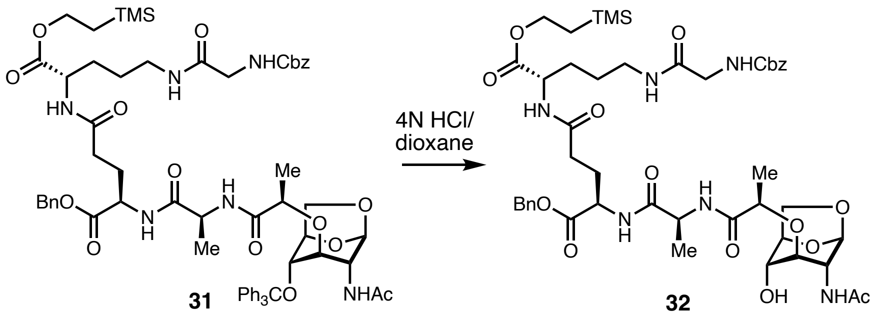

Benzyl N^2-(((R)-2-(((1R,2S,3R,4R,5R)-4-acetamido-2-hydroxy-6,8- dioxabicyclo [3.2.1]octan-3-yl)oxy)propanoyl)-L-alanyl)-N^5-((S)-5-(2-(((benzyloxy)carbonyl)amino) acetamido)-1-oxo-1-(2- (trimethylsilyl)ethoxy)pentan-2-yl)-D-glutaminate (**32**): **31** (100 mg, 0.082 mmol) was dissolved in DCM (4 mL) and was treated with 4N HCl/dioxane (0.103 mL) at 0 °C. The reaction mixture was warmed to RT, stirred for 3 h, and concentrated. The residue was purified by flash column chromatography (1-5% of 10% ACN/DCM) yielded **32** (60 mg, 75 %) as colorless solid. ^{1}H NMR (600 MHz, CD_3CN) δ 7.73 (d, *J* = 6.1 Hz, 1H), 7.38–7.26 (m, 10H), 7.20 (d, *J* = 8.0 Hz, 1H), 6.78 (s, 1H), 6.54 (d, *J* = 8.6 Hz, 1H), 6.06 (s, 1H), 5.31 (d, *J* = 1.7 Hz, 1H), 5.06 (d, *J* = 10.0 Hz), 4.47 (d, *J* = 5.6 Hz, 1H), 4.30 (ddd, *J* = 9.2, 8.0, 4.7 Hz, 1H), 4.23 (td, *J* = 8.6, 4.6 Hz, 1H), 4.19 – 4.02 (m, 4H), 4.05 (m, 1H), 3.83 (d, *J* = 8.7 Hz, 1H), 3.65 (dd, *J* = 7.8, 5.7 Hz, 3H), 3.61 (d, *J* = 4.6 Hz, 1H), 3.34 (p, *J* = 1.5 Hz, 1H), 3.14 (t, 2H), 2.41 (t, *J* = 7.6 Hz, 2H), 2.15 (m, 1H), 1.91–1.84 (m, 1H), 1.85 (s, 3H), 1.75 (m, 1H), 1.69–1.60 (m, 1H), 1.45 (p, *J* = 7.2 Hz, 2H), 1.38–1.23 (m, 6H), 0.95 (td, *J* = 8.0, 7.4, 4.9 Hz, 2H), 0 (s, 9H). ^{13}C NMR (101 MHz, CD_3CN) δ 173.53, 172.73, 172.62, 172.25, 171.33, 169.98, 169.38, 156.68, 137.12, 136.48, 128.53, 128.49, 128.09, 128.04, 127.96, 127.82, 117.38, 100.41, 79.19, 75.99, 75.46, 68.84, 66.32, 66.28, 65.96, 64.95, 63.26, 52.46, 52.39, 49.56, 48.95, 43.99, 38.19, 30.10, 28.09, 26.52, 25.55, 22.05, 17.78, 16.89, 16.49, 0.36, 0.19, 0.16. LRMS (ESI+) for $C_{46}H_{66}N_6O_{15}Si^+$ (971.146): 972.012 $[M+H]^+$.

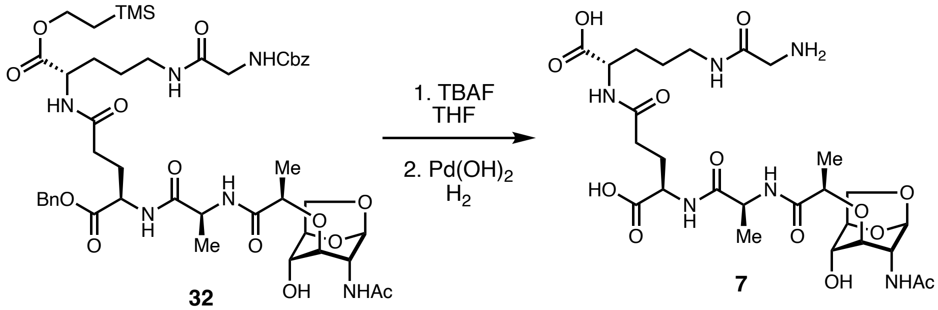

N^2-(((R)-2-(((1R,2S,3R,4R,5R)-4-acetamido-2-hydroxy-6,8- dioxabicyclo[3.2.1]octan-3-yl)oxy)propanoyl)-L-alanyl)-N^5-((S)-4-(2- aminoacetamido)-1-carboxybutyl)-D-glutamine (**7**): **32** (60 mg, 0.0391 mmol) was dissolved in THF (5 mL). TBAF (1M in THF, 156 uL, 0.156 mmol) was added. The reaction was stirred overnight, and condensed. The residue was dissolved in EtOAc and washed with 1N HCl (x3), dried (Na_2SO_4), condensed and placed under high vacuum for one hour. The crude product was dissolved in THF (1.2 mL), acetic acid (0.3 mL) and water (3 mL). 20% $Pd(OH)_2$ (13.5 mg, 0.0195 mmol) was added and the flask was evacuated three times and backfilled with hydrogen gas. After 20 h, the reaction mixture was filtered and condensed. The crude product was purified by Waters HPLC/MS using a preparative C18 column (0-45% acetonitrile in water with 0.1% formic acid over 4 mins, 20 mL/min) to yield **7** (14 mg, 53%) as white solid.^{1}H NMR (600 MHz, D_2O) δ 8.41 (s, 1H), 5.44 (d, *J* = 1.7 Hz, 1H), 4.64 (s, 1H), 4.36 (q, *J* = 7.1 Hz, 1H), 4.26 – 4.21 (m, 1H), 4.15 (dd, *J* = 7.4, 4.5 Hz, 2H), 4.10 (dd, *J* = 8.3, 4.8 Hz, 1H), 3.88 (d, *J* = 2.0 Hz, 1H), 3.84 (d, *J* = 1.9 Hz, 1H), 3.77 (dd, *J* = 7.8, 5.8 Hz, 1H), 3.73 (s, 2H), 3.39 (p, *J* = 1.6 Hz, 1H), 3.21 (t, *J* = 6.7 Hz), 2.27–2.20 (m, 2H), 2.07 (dt, *J* = 14.8, 7.5 Hz, 1H), 1.96 (s, 3H), 1.90 (dt, *J* = 14.9, 7.4 Hz, 1H), 1.75 (s, 1H), 1.62 (dq, *J* = 15.0, 7.8 Hz, 1H), 1.50 (h, *J* = 7.5 Hz, 2H), 1.39 (d, *J* = 7.2 Hz, 3H), 1.34 (d, *J* = 6.8 Hz, 3H).^{13}C NMR (151 MHz, D_2O) δ 178.86, 175.49, 174.86, 173.88, 173.62, 166.79, 99.97, 78.43, 75.92, 68.23, 65.22, 54.83, 54.58, 49.46, 40.46, 39.04, 32.17, 29.03, 28.07, 24.85, 21.83, 18.02, 17.16. HRMS *m/z*: $[M+H]^+$ calcd $C_{26}H_{43}N_6O_{13}^+$ 647.28881; Found 647.28814.

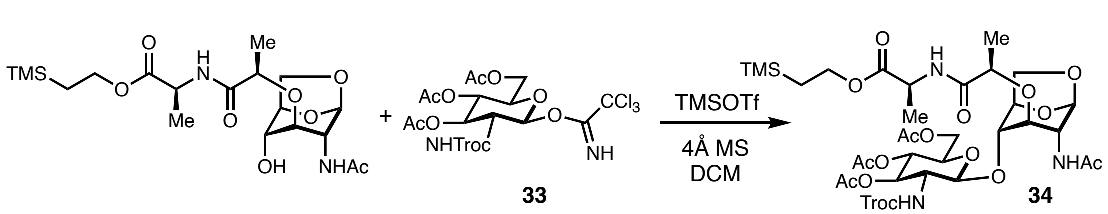

(2R,3S,4R,5R,6S)-6-(((1R,2S,3R,4R,5R)-4-acetamido-3-(((R)-1-oxo-1-(((S)-1-oxo-1- (2-(trimethylsilyl)ethoxy)propan-2-yl)amino)propan-2-yl)oxy)-6,8- dioxabicyclo [3.2.1]octan-2-yl)oxy)-2-(acetoxymethyl)-5-(((2,2,2- trichloroethoxy) carbonyl) amino)tetrahydro-2H-pyran-3,4-diyl diacetate (**34**): To a solution of glycosyl acceptor[2-3] (226 mg, 0.506 mmol) in DCM (10 mL) were added 4 Å molecular sieves (pre-activated, 1 g) and **33**[4] (0.98 g, 1.518 mmol). The reaction mixture was cooled to -78 °C and TMSOTf (19 ul, 0.101 mmol) was added dropwise. The solution was warmed slowly to RT and stirred for 24 h. The reaction mixture was filtered through Celite and washed with DCM. The organic layer was

washed with saturated aqueous $NaHCO_3$ solution and brine, dried (Na_2SO_4), and concentrated *in vacuo*. The residue was purified by flash column chromatography on silica gel (0-3% MeOH in 10% ACN/DCM to yield **34** (326 mg, 71 %) as a white foam. ^{1}H NMR (600 MHz, CD_3CN) δ 7.66 (d, *J* = 7.6 Hz, 1H), 6.39 (d, *J* = 9.5 Hz, 1H), 6.14 (d, *J* = 9.6 Hz, 1H), 5.25 (s, 1H), 5.14 (dd, *J* = 10.8, 9.3 Hz, 1H), 4.96 (t, *J* = 9.7 Hz, 1H), 4.87 (d, *J* = 12.2 Hz, 1H), 4.71 (d, *J* = 8.4 Hz, 1H), 4.58 (d, *J* = 12.2 Hz, 1H), 4.52 (d, *J* = 6.0 Hz, 1H), 4.31 (p, *J* = 7.3 Hz, 1H), 4.24 – 4.10 (m, 4H), 4.02 (dd, *J* = 12.3, 2.5 Hz, 1H), 3.97 (q, *J* = 6.7 Hz, 1H), 3.89 (d, *J* = 9.5 Hz, 1H), 3.81 – 3.72 (m, 2H), 3.67 (ddq, *J* = 17.7, 9.2, 5.2, 4.1 Hz, 2H,), 3.44 – 3.40 (m, 1H), 2.11 (s, 6H), 1.97 (s, 3H), 1.93 (s, 3H), 1.32 (d, *J* = 7.3 Hz, 3H), 1.27 (t, *J* = 7.5 Hz, 3H), 0.95 (ddd, *J* = 10.4, 6.8, 1.9 Hz, 2H), 0.00 (s, 9H). ^{13}C NMR (151 MHz, CD_3CN) δ 173.10, 172.52, 170.78, 170.70, 170.07, 170.04, 155.85, 101.21, 100.00, 78.32, 76.43, 75.37, 74.65, 73.72, 72.13, 72.09, 69.10, 65.01, 63.61, 62.36, 56.60, 48.34, 48.29, 22.42, 20.49, 20.43, 20.39, 20.36, 17.96, 17.42, 17.30, 17.29, -2.00. LRMS (ESI+) for $C_{34}H_{52}Cl_3N_3O_{17}Si^+$ (907.21): 908.16 $[M+H]^+$.

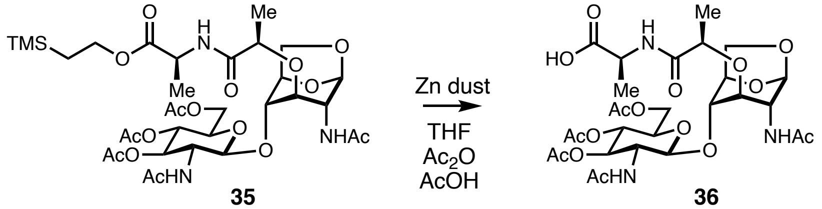

((R)-2-(((1R,2S,3R,4R,5R)-4-acetamido-2-(((2S,3R,4R,5S,6R)-3-acetamido-4,5-diacetoxy-6-(acetoxymethyl)tetrahydro-2H-pyran-2-yl)oxy)-6,8- dioxabicyclo[3.2.1]octan-3-yl)oxy)propanoyl)-L-alanine (**36**): **34** (0.35 g, 0.386 mmol) was dissolved in THF/Ac2O/AcOH (3:2:1, 60 mL). Zn dust (992 mg, 15.4 mmol) was added and stirred overnight. The reaction mixture was filtered through Celite, washed with DCM, and concentrated. The resulting residue was co-evaporated with toluene (x3) and then dissolved in DCM. The organic layer was washed with saturated aqueous $NaHCO_3$ and brine, dried (Na_2SO_4), filtered, and concentrated *in vacuo*. Flash column chromatography on silica gel (3-5% MeOH in 10% ACN/DCM) yielded **35** (278 mg, 93 %) as a white foam. Subsequently, **35** was dissolved in THF (10 mL). TBAF (1M in THF, 718 uL, 0.718 mmol) was added. The reaction mixture was stirred overnight then condensed. The residue was dissolved in EtOAc and washed 1N HCl (x3), dried (Na_2SO_4), filtered and condensed (under high vacuum for 1 h). The crude product was purified using a silica plug (10% MeOH/DCM with 1% acetic acid) to remove residual TBAF yielding **36** (214 mg, 89 %) as a white foam. ^{1}H NMR (600 MHz, $CDCl_3$) δ 7.79 (d, *J* = 8.0 Hz, 1H), 6.78 (d, *J* = 10.0 Hz, 1H), 6.07 (dd, *J* = 8.9, 2.7 Hz, 1H), 5.30 (d, *J* = 2.0 Hz, 1H), 5.09 – 5.02 (m, 2H), 4.52 (q, *J* = 7.4 Hz, 1H), 4.50–4.45 (m, 2H), 4.31 (d, *J* = 7.5 Hz, 1H), 4.25 (dd, *J* = 12.4, 5.1 Hz, 1H), 4.21–4.10 (m, 3H), 4.12–4.03 (m, 2H), 3.77 (dd, *J* = 7.5, 5.7 Hz, 1H), 3.73 (d, *J* = 1.9 Hz, 1H), 3.64 (td, *J* = 5.5, 2.7 Hz, 1H), 3.50 (t, *J* = 1.6 Hz, 1H), 2.08 (s, 3H), 2.05 (s, 3H), 2.01 (d, *J* = 8.0 Hz, 6H), 1.96 (s, 3H), 1.36 (dd, *J* = 21.2, 6.9 Hz, 6H), 0.99 – 0.92 (m, 2H). ^{13}C NMR (151 MHz, $CDCl_3$) δ 174.68, 173.42, 173.27, 173.00, 172.37, 172.10, 170.74, 102.81, 101.48, 78.98, 77.23, 75.82, 74.10, 73.64, 73.35, 69.77, 66.01, 65.08, 63.53, 55.64, 49.30, 48.58, 25.09, 24.23, 22.22, 22.17, 22.08, 19.70, 18.80, 18.76, 0.00. LRMS (ESI+) for $C_{28}H_{41}N_3O_{16}$ (675.25): 676.32 $[M+H]^+$.

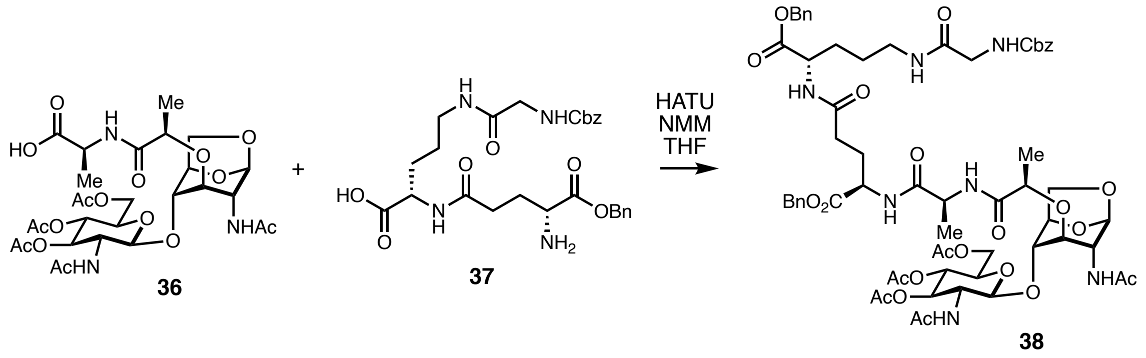

(2R,3S,4R,5R,6S)-5-acetamido-6-(((1R,2S,3R,4R,5R)-4-acetamido-3-(((11S,16R,19S,22R)-16-((benzyloxy)carbonyl)-19-methyl-3,6,13,18,21-pentaoxo- 1-phenyl-11-((2-(trimethylsilyl)ethoxy)carbonyl)-2-oxa-4,7,12,17,20- pentaazatricosan-22-yl)oxy)-6,8-dioxabicyclo[3.2.1]octan-2-yl)oxy)-2- (acetoxymethyl)tetrahydro-2H-pyran-3,4-diyl diacetate (**38**): **37** (440 mg, 0.65 mmol) was dissolved in THF (5 mL) H-D-Glu(OBn)-L-Orn(Gly(Z))-TMSE (1.13 g, 1.3 mmol, 2 eq) was added. NMM (214 ul, 1.95 mmol) and HATU (494 mg, 1.3 mmol) were added and the reaction was stirred overnight and then condensed. The residue was dissolved in EtOAc, washed with brine, dried (Na_2SO_4), filtered, and concentrated *in vacuo*. Flash column chromatography (4–6% MeOH in 10% ACN/DCM) yielded **38** (650 mg, 76 %) as a colorless solid. ^1H NMR (600 MHz, MeOD) δ 7.31 (dt, J = 8.8, 4.3 Hz, 7H), 7.30–7.22 (m, 3H), 5.29 (d, J = 2.0 Hz, 1H,), 5.16 (dd, J = 10.9, 9.2 Hz, 1H), 5.12 (s, 2H), 5.06 (s, 2H), 4.96 (t, J = 9.7 Hz, 1H), 4.68 (d, J = 8.4 Hz, 1H), 4.55 (s, 1H), 4.43–4.37 (m, 2H), 4.31–4.22 (m, 2H), 4.16 (t, J = 8.5 Hz, 2H), 4.09 (td, J = 6.6, 3.6 Hz, 2H), 3.98 (dd, J = 10.7, 8.4 Hz, 1H), 3.91 (s, 1H), 3.81–3.75 (m, 1H), 3.70 (t, J = 3.3 Hz, 3H), 3.56–3.51 (m, 1H), 3.19–3.14 (m, 2H), 2.28 (t, J = 7.6 Hz, 2H), 2.16 (dt, J = 13.7, 6.7 Hz, 1H), 2.03 – 1.98 (m, 6H), 1.96 (d, J = 2.8 Hz, 6H), 1.93 (d, J = 1.4 Hz, 4H), 1.78 (dd, J = 14.0, 6.7 Hz, 1H), 1.61 (s, 1H), 1.52 (tt, J = 13.8, 7.3 Hz, 2H), 1.33 (dd, J = 10.3, 6.9 Hz, 6H), 0.97 (t, J = 8.5 Hz, 2H), -0.00 (s, 9H). ^{13}C NMR (151 MHz, MeOD) δ 173.51, 173.41, 173.36, 173.15, 172.33, 171.81, 171.24, 170.86, 170.46, 169.85, 157.62, 136.69, 135.81, 128.20, 128.11, 127.91, 127.83, 127.69, 127.56, 100.56, 100.09, 77.73, 75.81, 75.33, 73.37, 72.03, 71.65, 68.68, 66.63, 66.50, 64.49 (6-C), 63.26, 61.71, 53.77, 52.48, 52.10, 48.72, 48.03, 43.65, 38.43, 31.35, 28.34, 26.79, 25.56, 21.79, 21.22, 19.30, 19.18, 19.14, 17.18, 16.95, 16.85, -2.88. LRMS (ESI+) for $C_{60}H_{85}N_7O_{23}Si^+$ (1299.55): 1301.01 $[M+H]^+$.

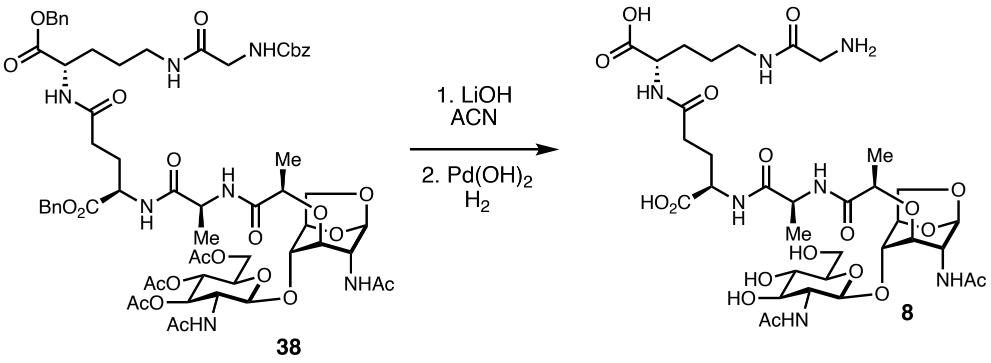

N^2-(((R)-2-(((1R,2S,3R,4R,5R)-4-acetamido-2-(((2S,3R,4R,5S,6R)-3-acetamido- 4,5-dihydroxy-6-(hydroxymethyl)tetrahydro-2H-pyran-2-yl)oxy)-6,8- dioxabicyclo[3.2.1] octan-3-yl)oxy)propanoyl)- L-alanyl)-N^5-((S)-4-(2- aminoacetamido)-1-carboxybutyl)-D-glutamine (**8**) : **38** (0.037 g, 0.0288 mmol) was dissolved in acetonitrile (1.44 mL). A 0.1 M LiOH solution (1.44 mL) was added dropwise at 0 °C, and slowly warmed to RT. The reaction stirred overnight. Reaction progress was monitored by LCMS and once complete, the reaction was quenched with acetic acid (pH ~7), condensed and placed on a high vacuum for 12 h. The crude product was re-suspended in THF (800 uL), water (2.45 mL) and acetic acid (210 uL). 20% $Pd(OH)_2/C$ (10 mg, 0.0144 mmol, 0) was added, and the flask was evacuated and backfilled with hydrogen gas. After stirring overnight, filtered and condensed. The residue was purified via reverse phase SunFire Prep C18 column using an Autopure (0-20% acetonitrile in water with 0.1% Formic acid over 5 min) to yield **8** (13 mg, 53%) as a white powder. ^{1}H NMR (600 MHz, D_2O) δ 5.40 (s, 1H), 4.66 (d, *J* = 6.1 Hz, 1H), 4.62 (d, *J* = 8.4 Hz, 1H), 4.34 (q, *J* = 7.0 Hz, 1H), 4.23 (d, *J* = 7.9 Hz, 1H), 4.14 (q, *J* = 6.7 Hz, 1H), 3.94 (d, *J* = 14.0 Hz, 2H), 3.85 (d, *J* = 12.2 Hz, 1H), 3.79 – 3.68 (m, 5H), 3.57–3.50 (m, 2H), 3.45–3.37 (m, 2H), 3.22 (t, *J* = 6.5 Hz, 2H), 2.30 (d, *J* = 8.0 Hz, 2H), 2.18–2.09 (m, 1H), 2.02 (d, *J* = 11.6 Hz, 6H), 1.97 (s, 1H, CH2*CH2*CH(Glu)), 1.82 (s, 1H), 1.68 (s, 1H), 1.54 (d, *J* = 8.7 Hz, 2H), 1.38 (d, *J* = 6.9 Hz, 3H), 1.34 (d, *J* = 6.7 Hz, 3H). ^{13}C NMR (151 MHz, D_2O) δ 175.44, 175.02, 174.41, 173.70, 166.76, 163.28, 163.05, 162.81, 100.59, 99.91, 77.20, 76.11, 76.01, 74.44, 73.61, 73.34, 69.82, 64.82, 60.63, 55.54, 49.43, 48.86, 40.40, 38.87, 31.76, 28.14, 26.97 24.83, 22.35, 21.93, 18.00, 16.99. HRMS *m/z*: $[M+H]^+$ calcd $C_{34}H_{56}N_7O_{18}^+$ 850.36036; Found 850.36544.

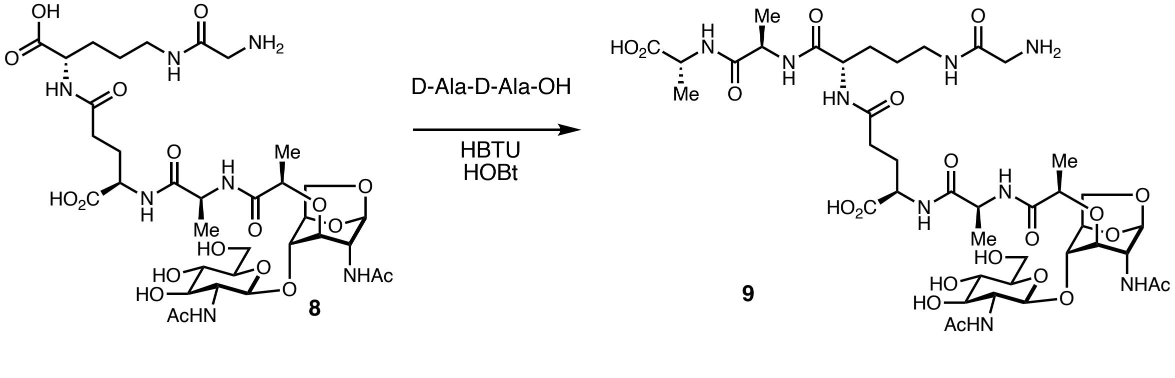

Compound **9**: To a solution of **8** (2 mg, 0.0024 mmol) in DMF (600 uL) was added HBTU (1.5 mg, 0.006 mmol) and HOBt (1 mg, 0.006 mmol), and the solution was cooled to 0 °C and stirred for 15 min. D-Ala-D-Ala-OH (1.4 mg, 0.006 mmol) was added, and the solution was stirred for 1 h at 0 °C and then at RT overnight. Reaction progress was monitored by LCMS. Once complete, the reaction mixture was filtered through a small plug of Celite and then condensed. The resulting oil was dissolved in water and purified by reverse phase HPLC (0-20% acetonitrile in water with 0.01% TFA). ^{1}H NMR (400 MHz, MeOD) δ 5.53 (s, 1H), 4.66 (s, 1H), 4.58 – 4.39 (m, 3H), 4.10 (dd, *J* = 9.4, 4.2 Hz, 2H), 3.96 (dd, *J* = 10.4, 3.7 Hz, 2H), 3.85 (t, *J* = 9.7 Hz, 2H), 3.81 – 3.64 (m, 4H), 3.59 (s, 2H), 3.50 (t, *J* = 9.0 Hz, 2H), 3.38 (td, *J* = 10.3, 5.2 Hz, 3H), 3.07 (d, *J* = 9.5 Hz, 1H), 2.19 – 2.03 (m, 2H), 1.96 (d, J = 10.5 Hz, 2H), 1.88 (s, 4H), 1.82 (s, 16H), 1.64 (dt, *J* = 13.4, 3.8 Hz, 5H), 1.58 – 1.43 (m, 6H), 1.41 – 1.25 (m, 10H), 1.23 (d, *J* = 5.6 Hz, 9H), 1.20 – 0.98 (m, 10H), 0.83 (t, *J* = 6.7 Hz, 3H). HRMS *m/z*: $[M+H]^+$ calcd $C_{40}H_{65}N_9O_{20}^+$ 991.43459; Found 991.43465.

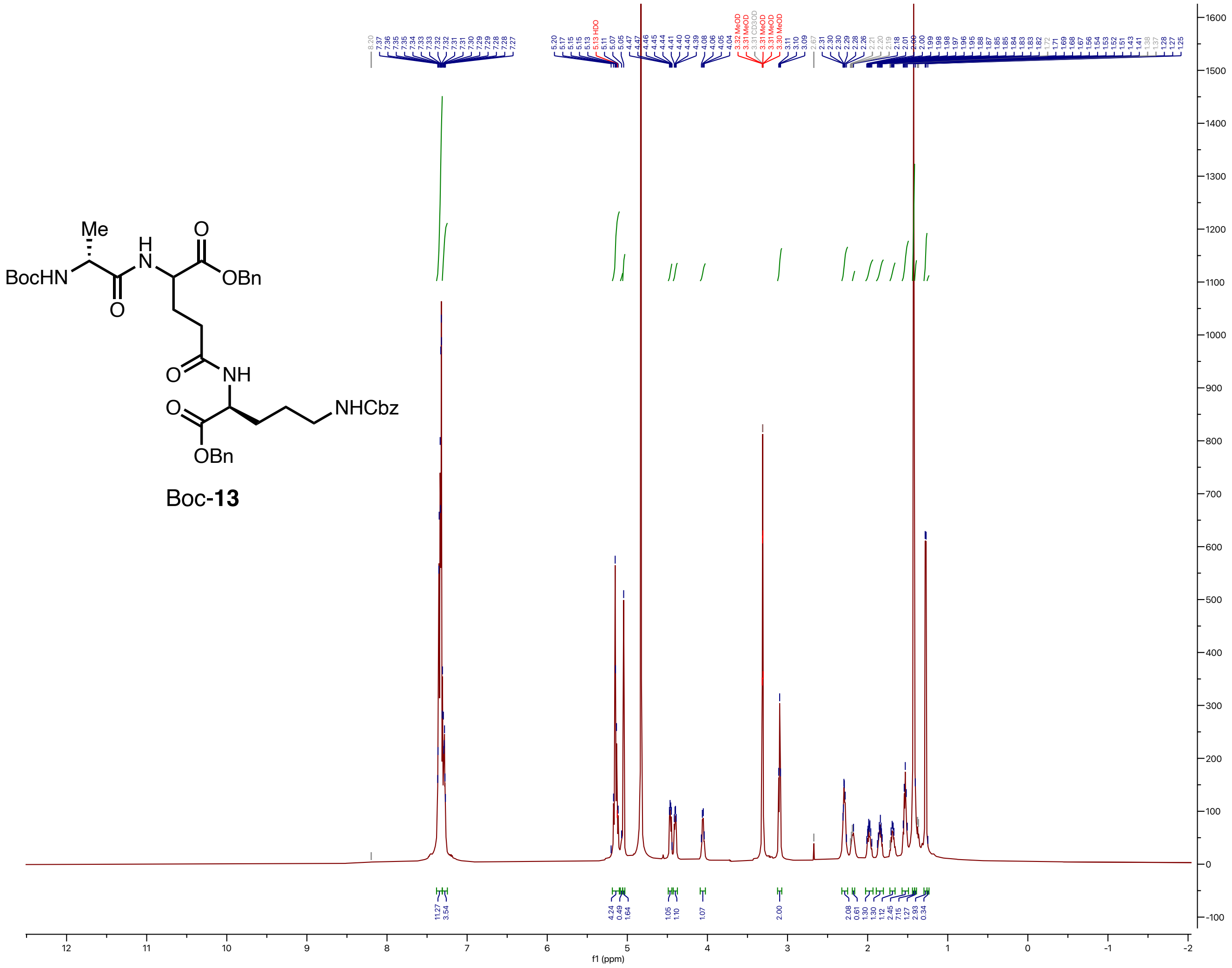

Boc-**13**

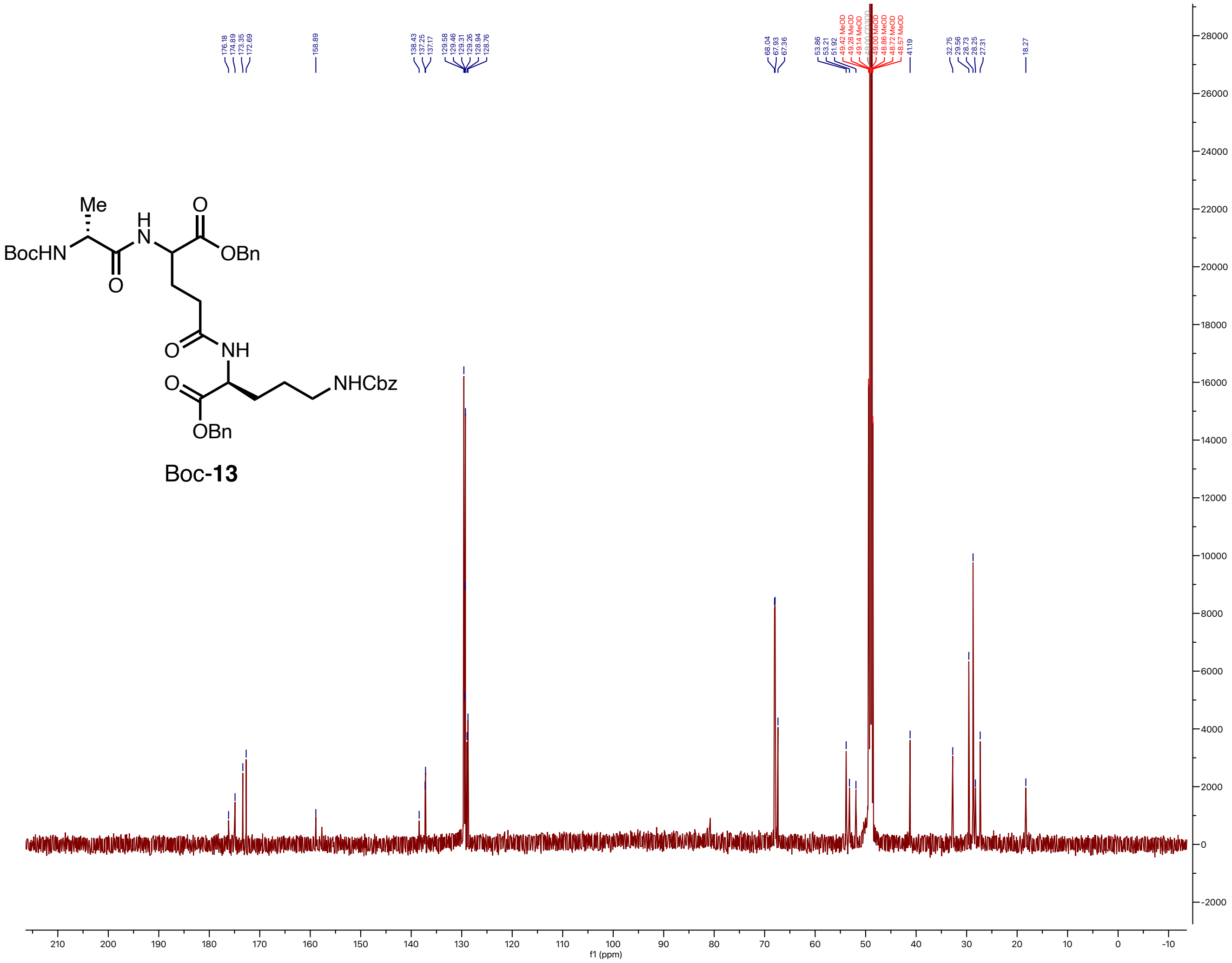
Me
H
N
O
BocHN
OBn
O
O
NH
O
NHCbz
OBn
176.18
174.89
173.35
172.69
158.89
138.43
137.25
137.17
129.58
129.46
129.31
129.26
128.94
128.76
68.04
67.93
67.36
53.86
53.21
51.92
49.42 MeOD
49.28 MeOD
49.14 MeOD
48.86 MeOD
48.72 MeOD
48.57 MeOD
41.19
32.75
29.56
28.73
28.26
27.31
18.27
f1 (ppm)

Boc-**13**

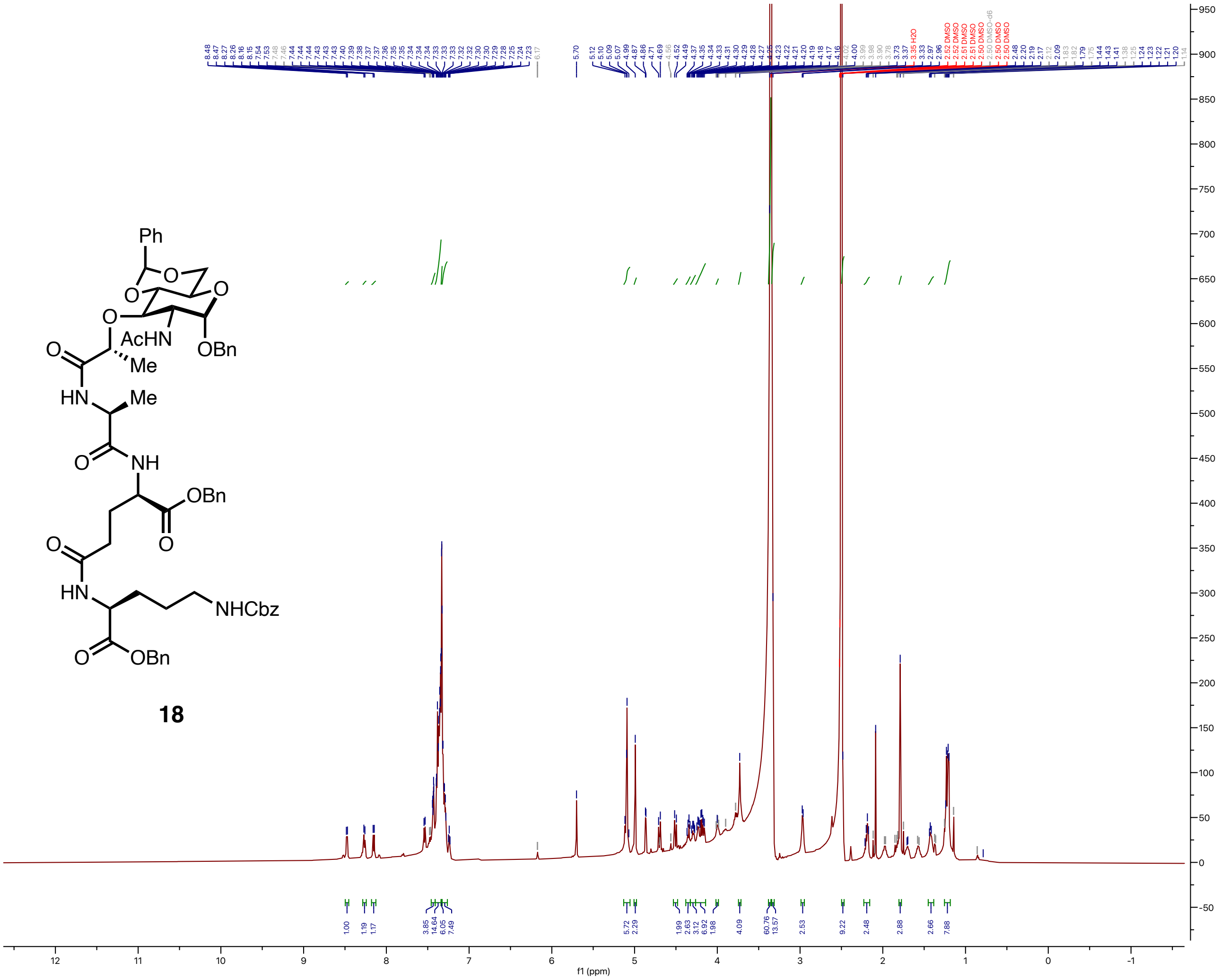

18

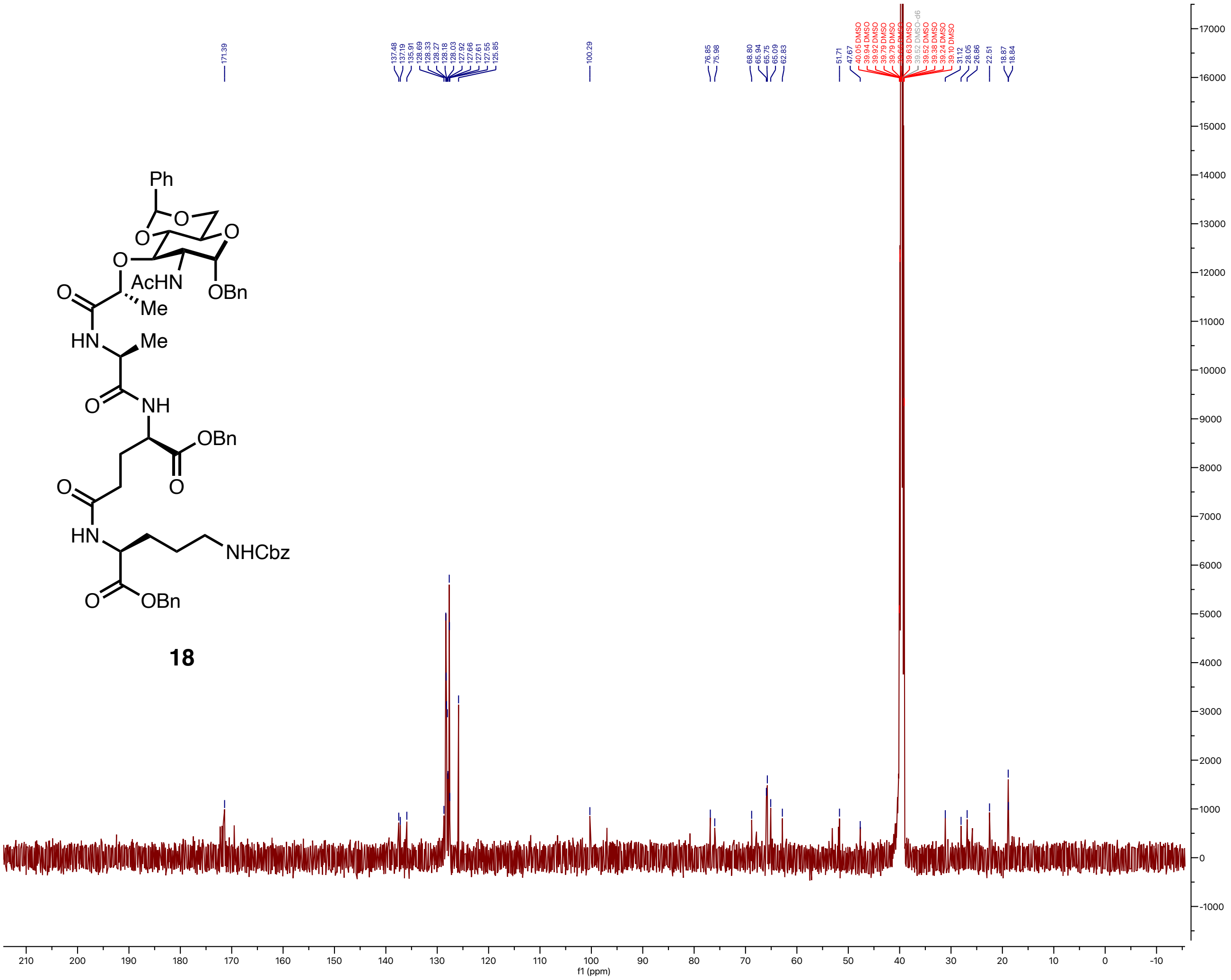

18

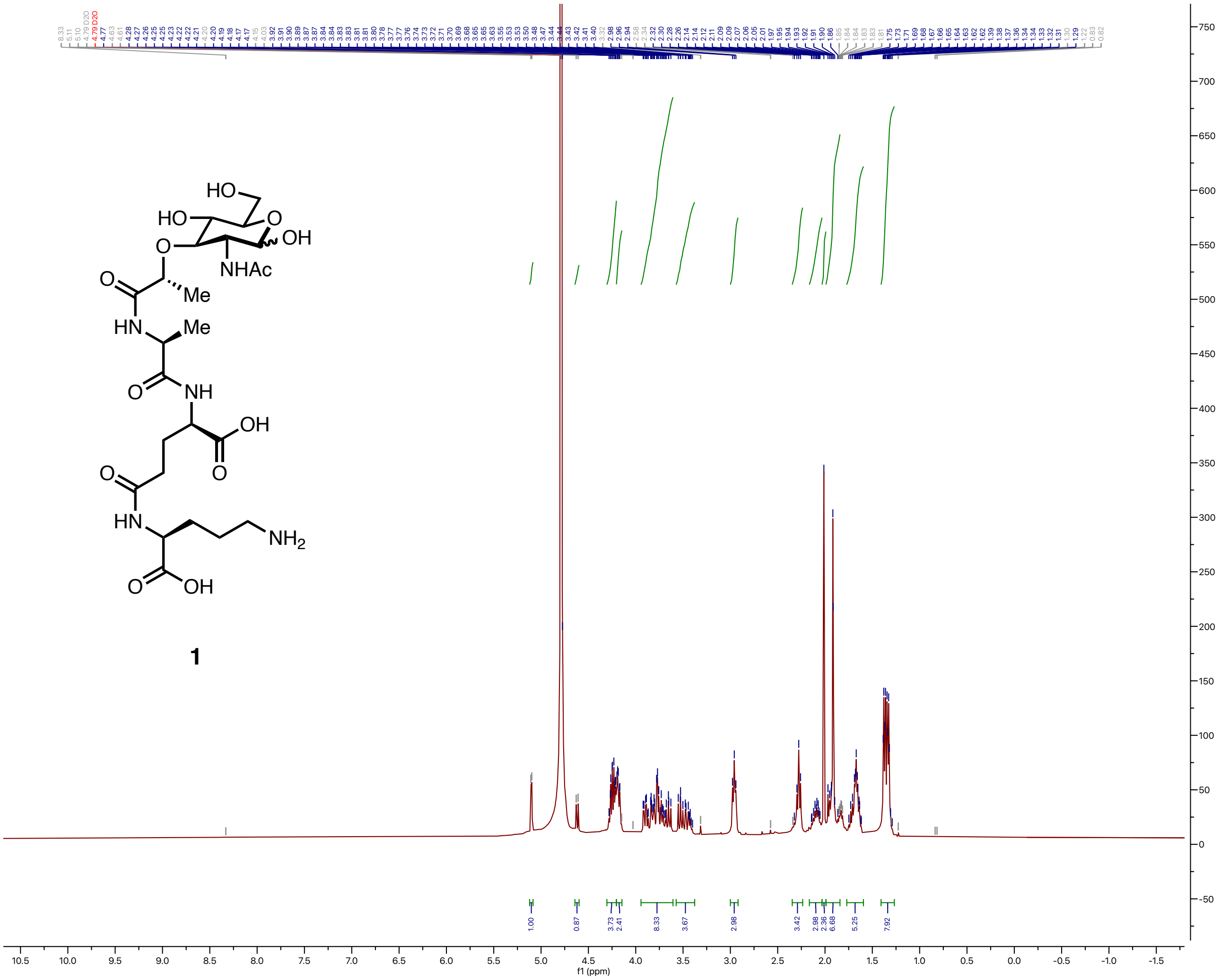

HO
HO
O
OH
NHAc
O
Me
HN
Me
O
NH
OH
O
O
HN
NH2
O
OH
1
f1 (ppm)

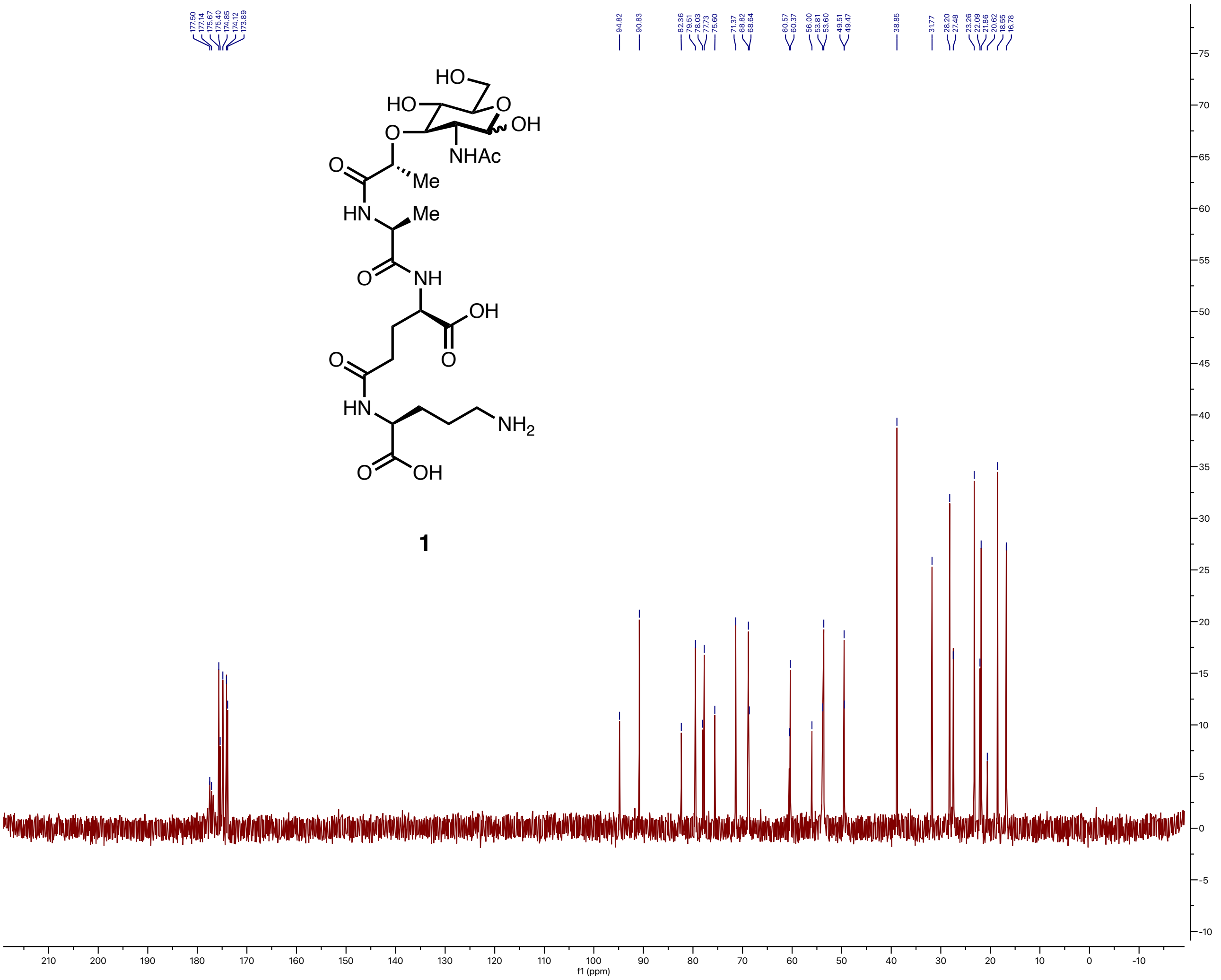

177.50
177.14
175.67
175.40
174.85
174.12
173.89
94.82
90.83
82.36
79.51
78.03
77.73
75.60
71.37
68.82
68.64
60.57
60.37
56.00
53.81
53.60
49.51
49.47
38.85
31.77
28.20
27.48
23.26
22.09
21.86
20.62
18.55
16.78
HO
HO
O
OH
NHAc
O
O
Me
HN
Me
O
NH
OH
O
O
HN
NH2
O
OH
1
f1 (ppm)

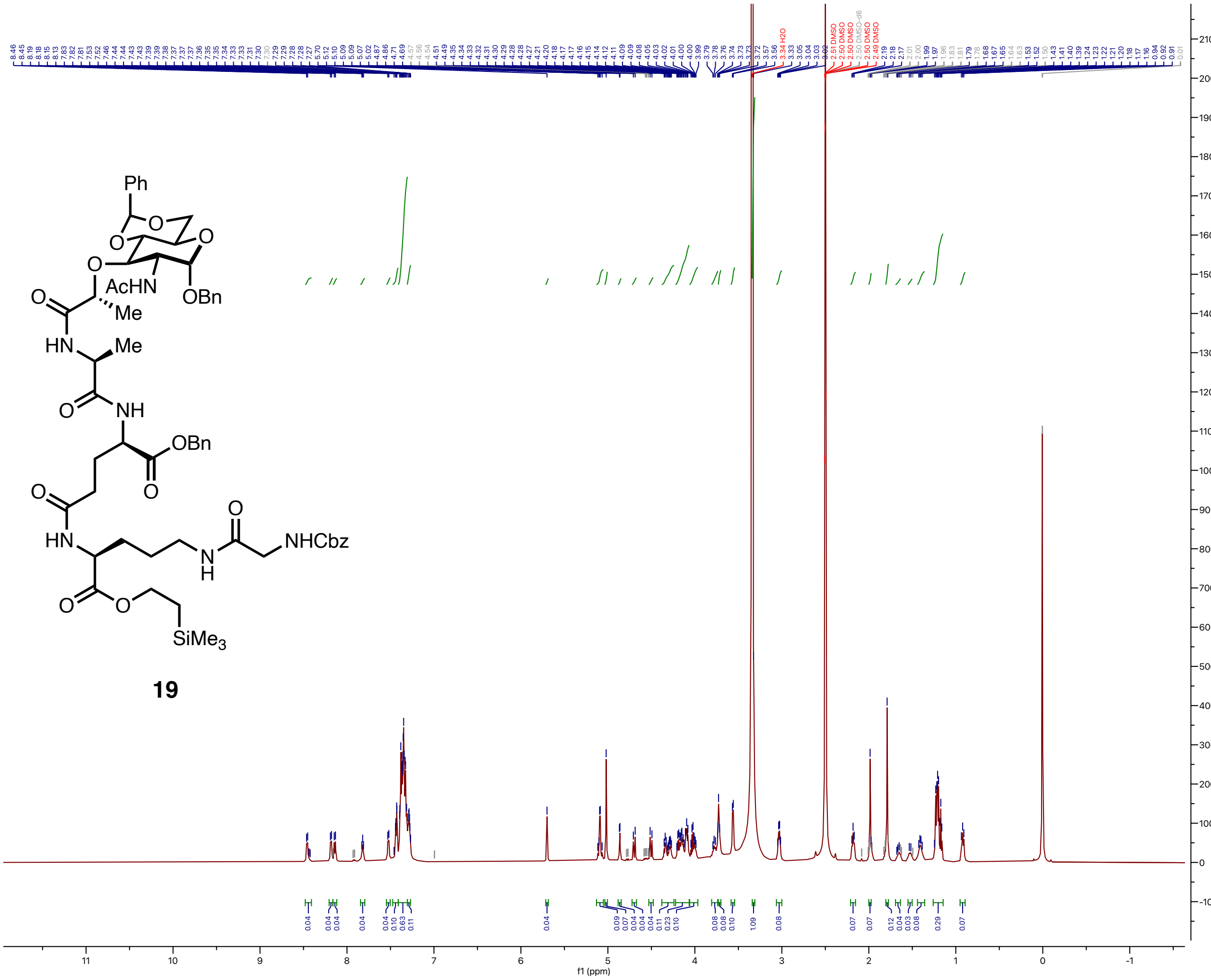
19
f1 (ppm)

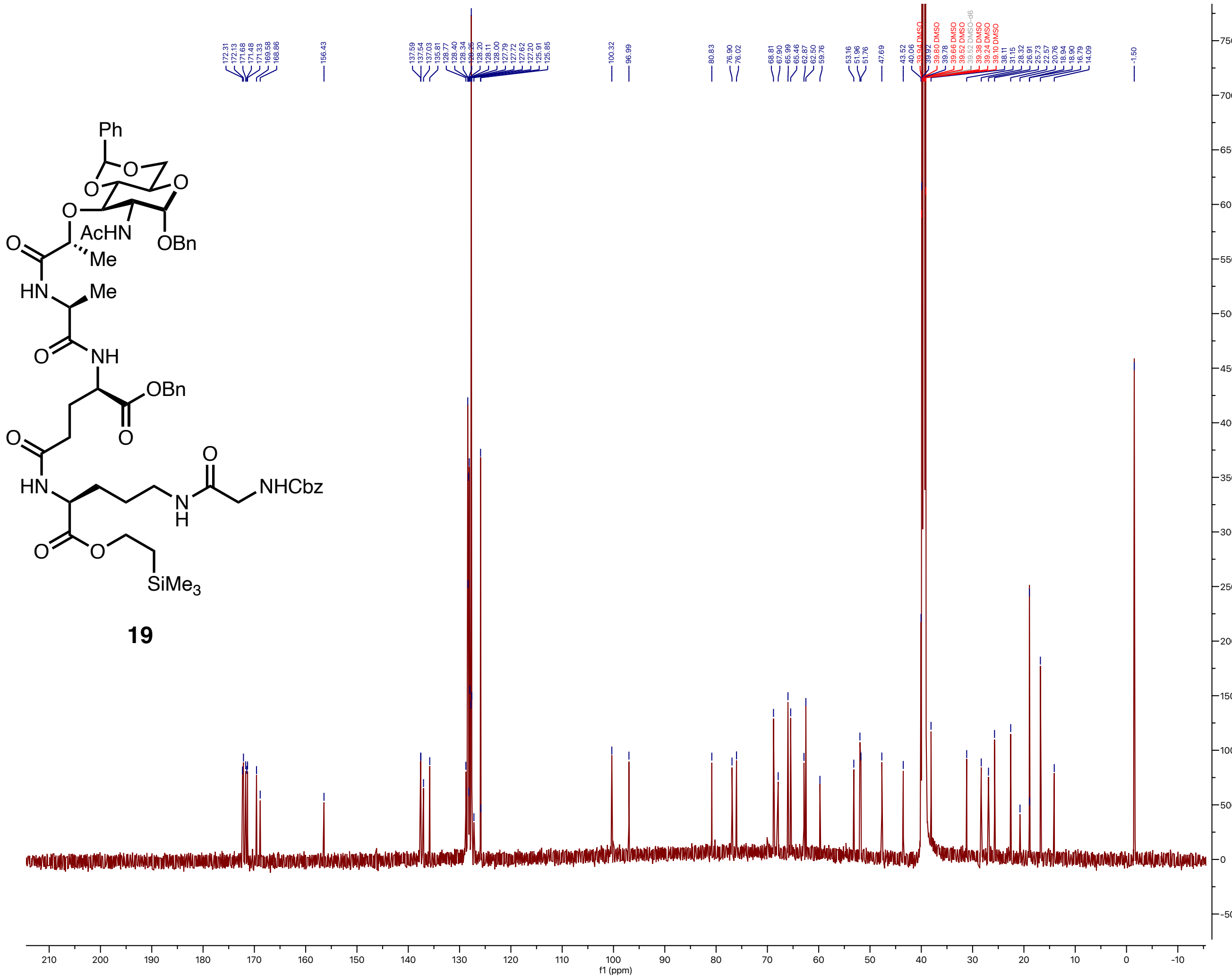
f1 (ppm)

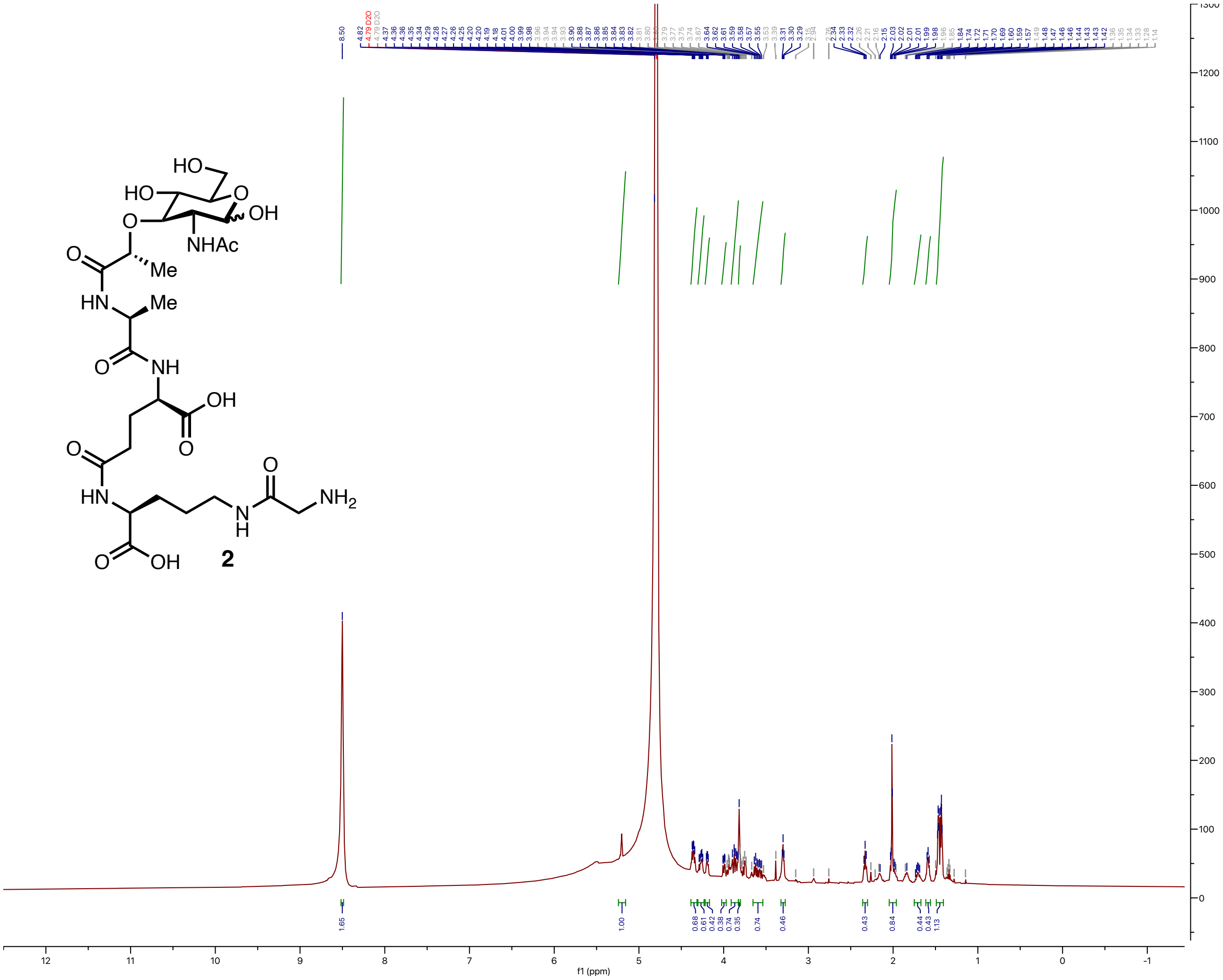

2

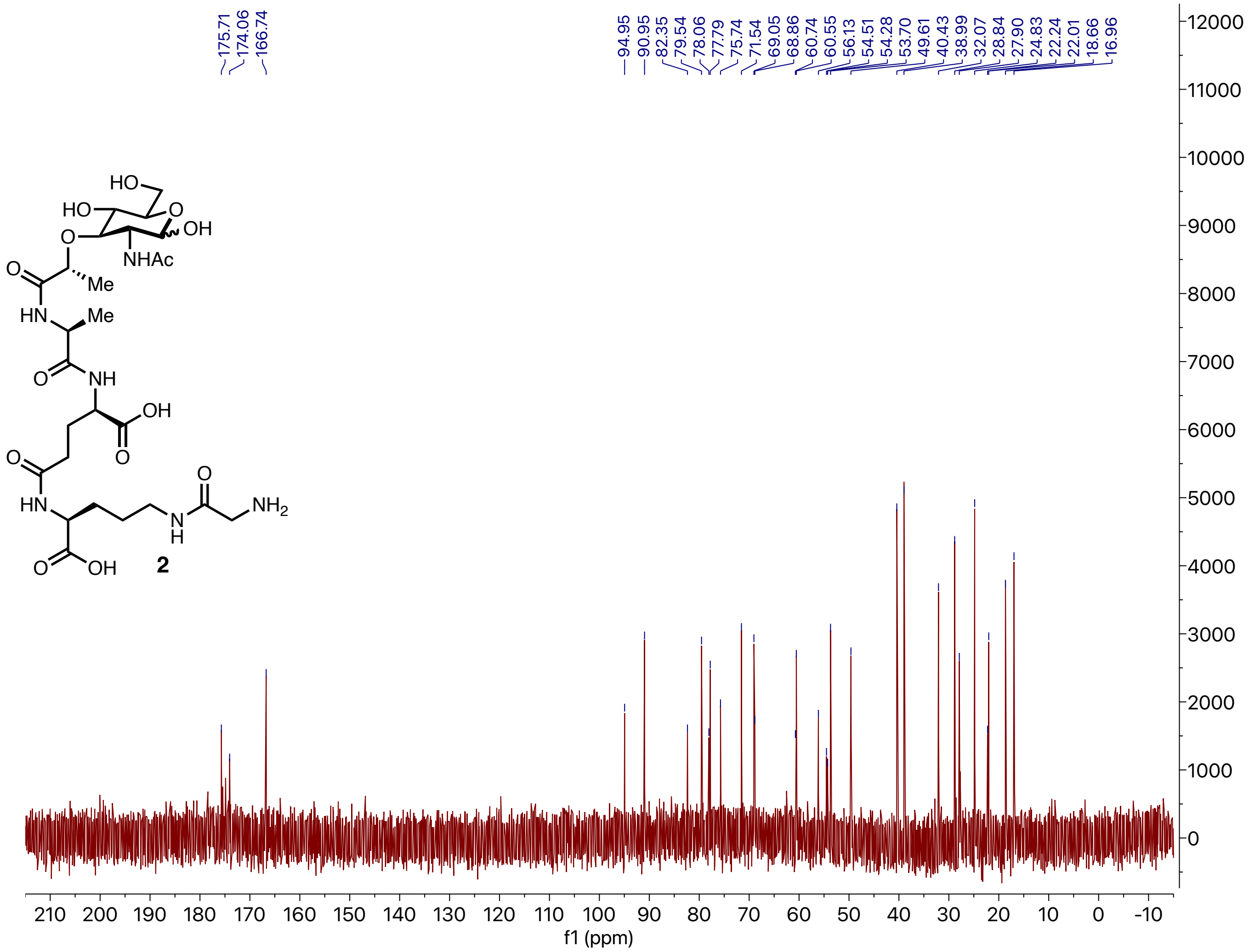
HO
HO
O
OH
O
NHAc
O
Me
HN
Me
O
NH
OH
O
O
HN
O
N
H
NH2
O
OH
2
175.71
174.06
166.74
94.95
90.95
82.35
79.54
78.06
77.79
75.74
71.54
69.05
68.86
60.74
60.55
56.13
54.51
54.28
53.70
49.61
40.43
38.99
32.07
28.84
27.90
24.83
22.24
22.01
18.66
16.96
f1 (ppm)

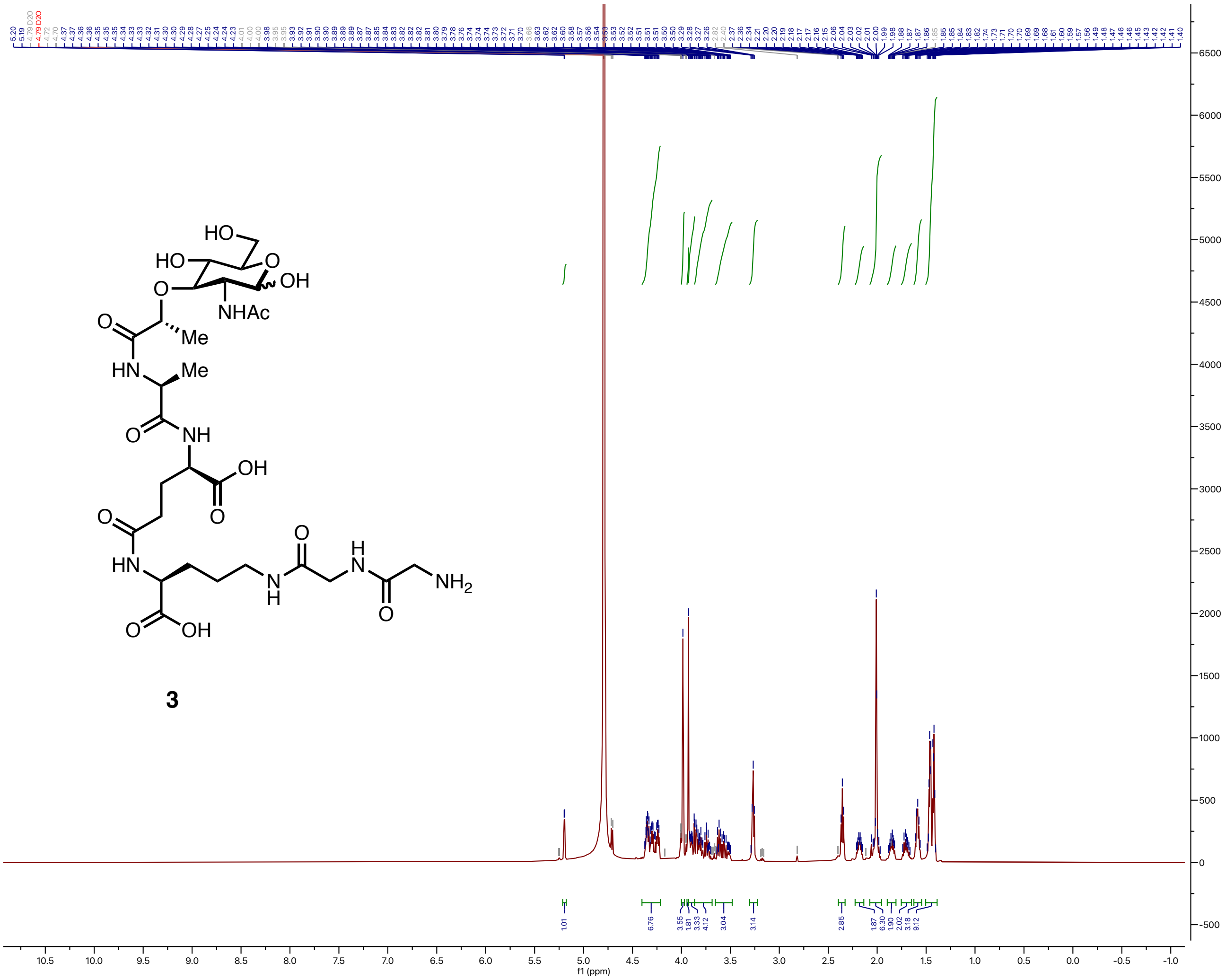
3
f1 (ppm)

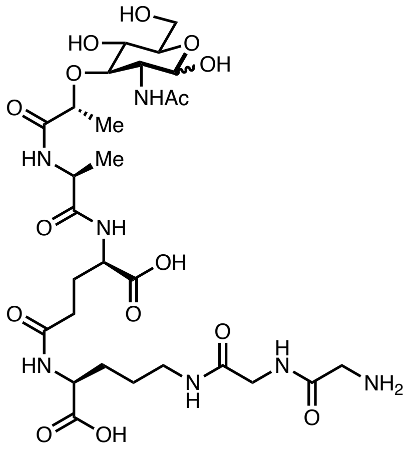

3

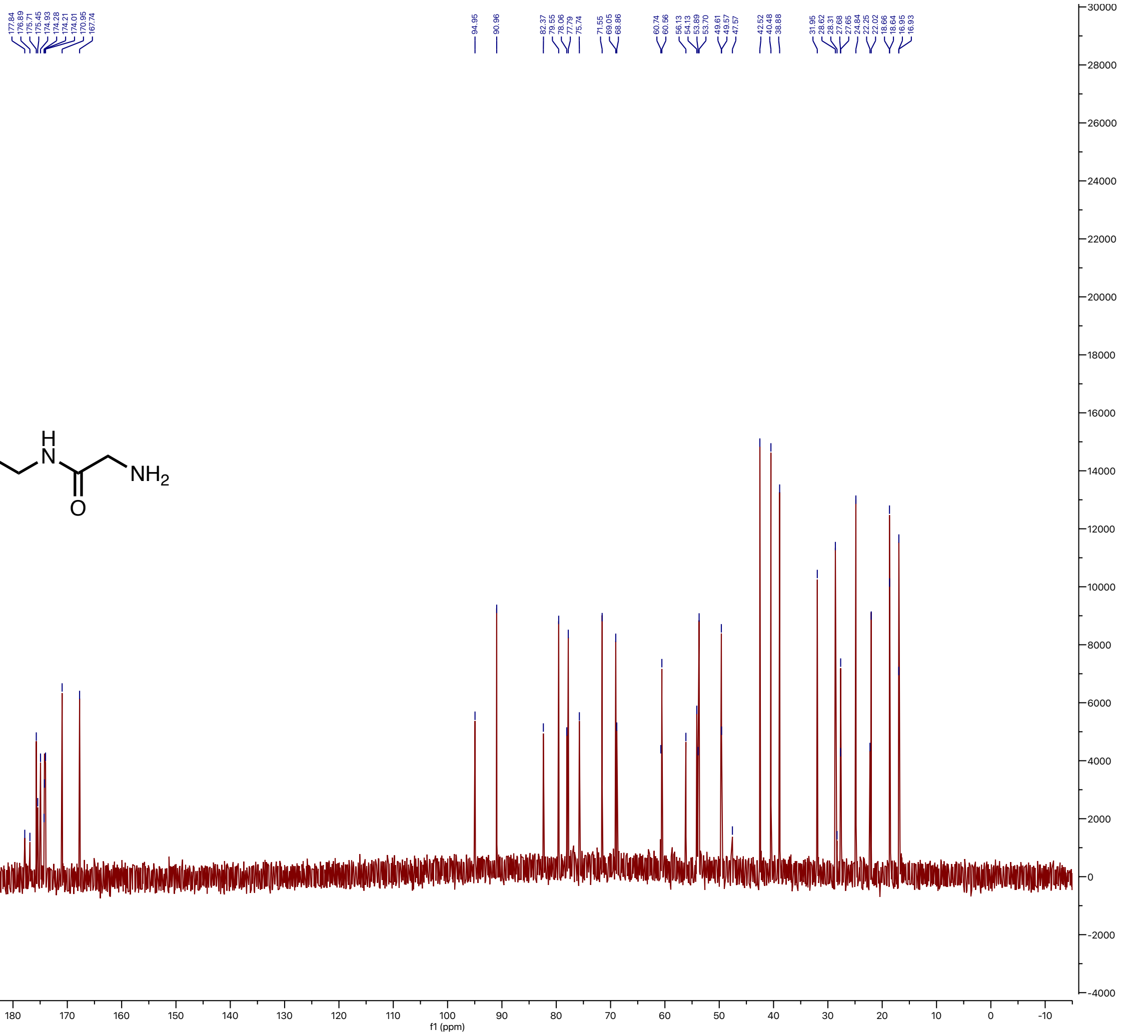

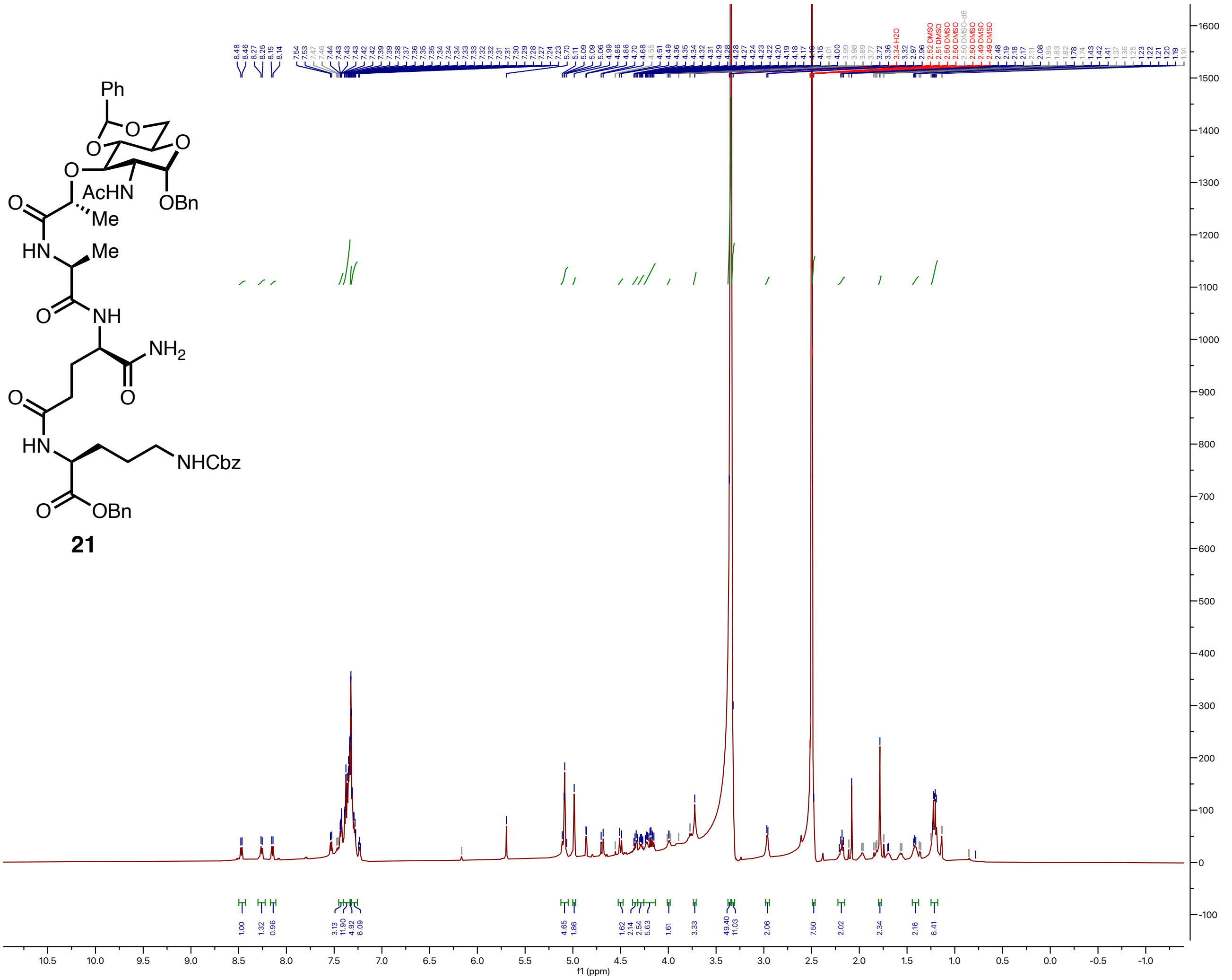

Ph
O
O
O
O
AcHN
OBn
O
Me
HN
Me
O
NH
NH2
O
O
HN
NHCbz
O
OBn
21
f1 (ppm)

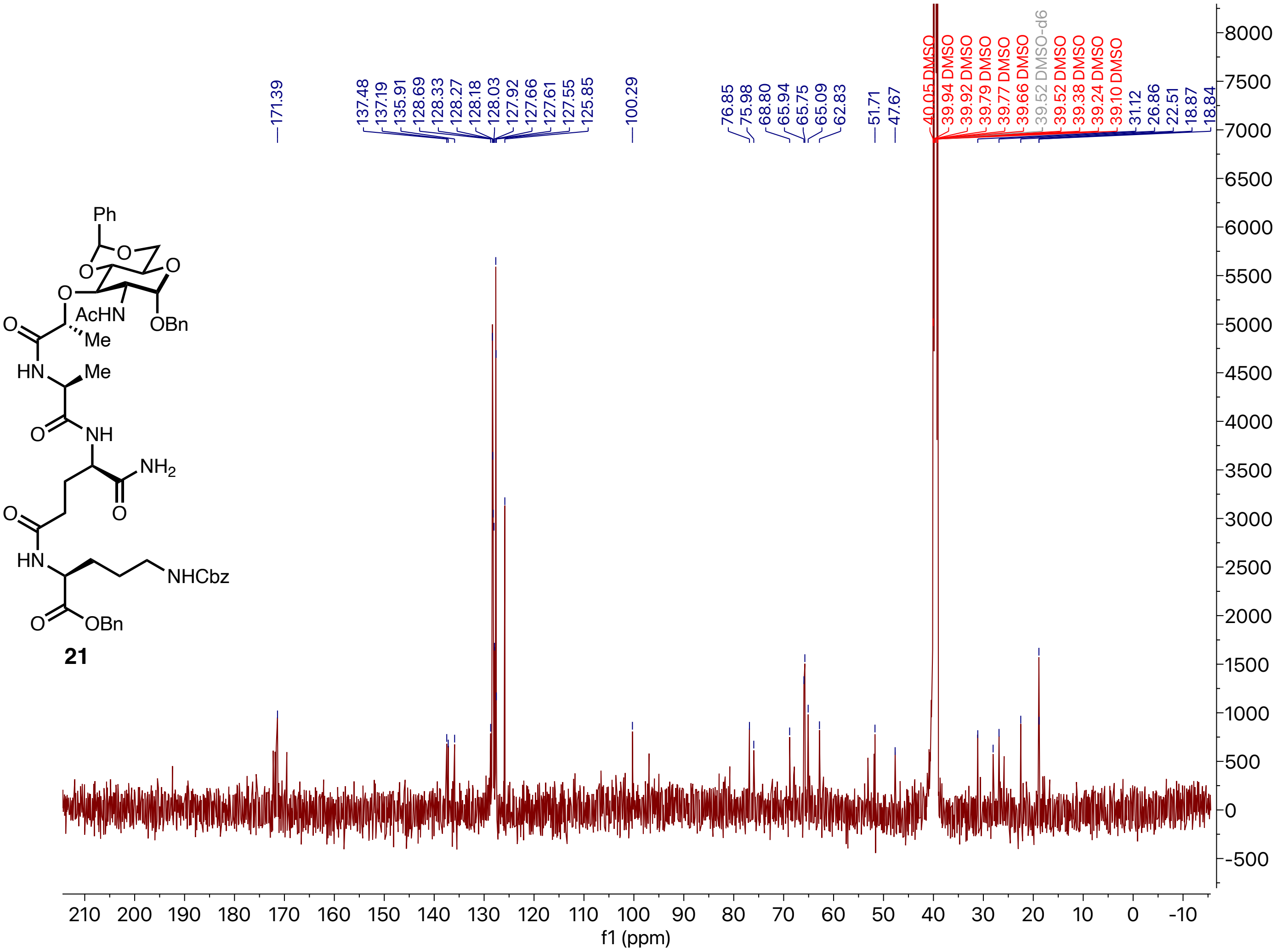

Ph
O
O
O
O
AcHN
OBn
O
Me
HN
Me
O
NH
NH2
O
O
HN
NHCbz
O
OBn

21

171.39
137.48
137.19
135.91
128.69
128.33
128.27
128.18
128.03
127.92
127.66
127.61
127.55
125.85
100.29
76.85
75.98
68.80
65.94
65.75
65.09
62.83
51.71
47.67
40.05 DMSO
39.94 DMSO
39.92 DMSO
39.79 DMSO
39.77 DMSO
39.66 DMSO
39.52 DMSO-d6
39.52 DMSO
39.38 DMSO
39.24 DMSO
39.10 DMSO
31.12
26.86
22.51
18.87
18.84
f1 (ppm)

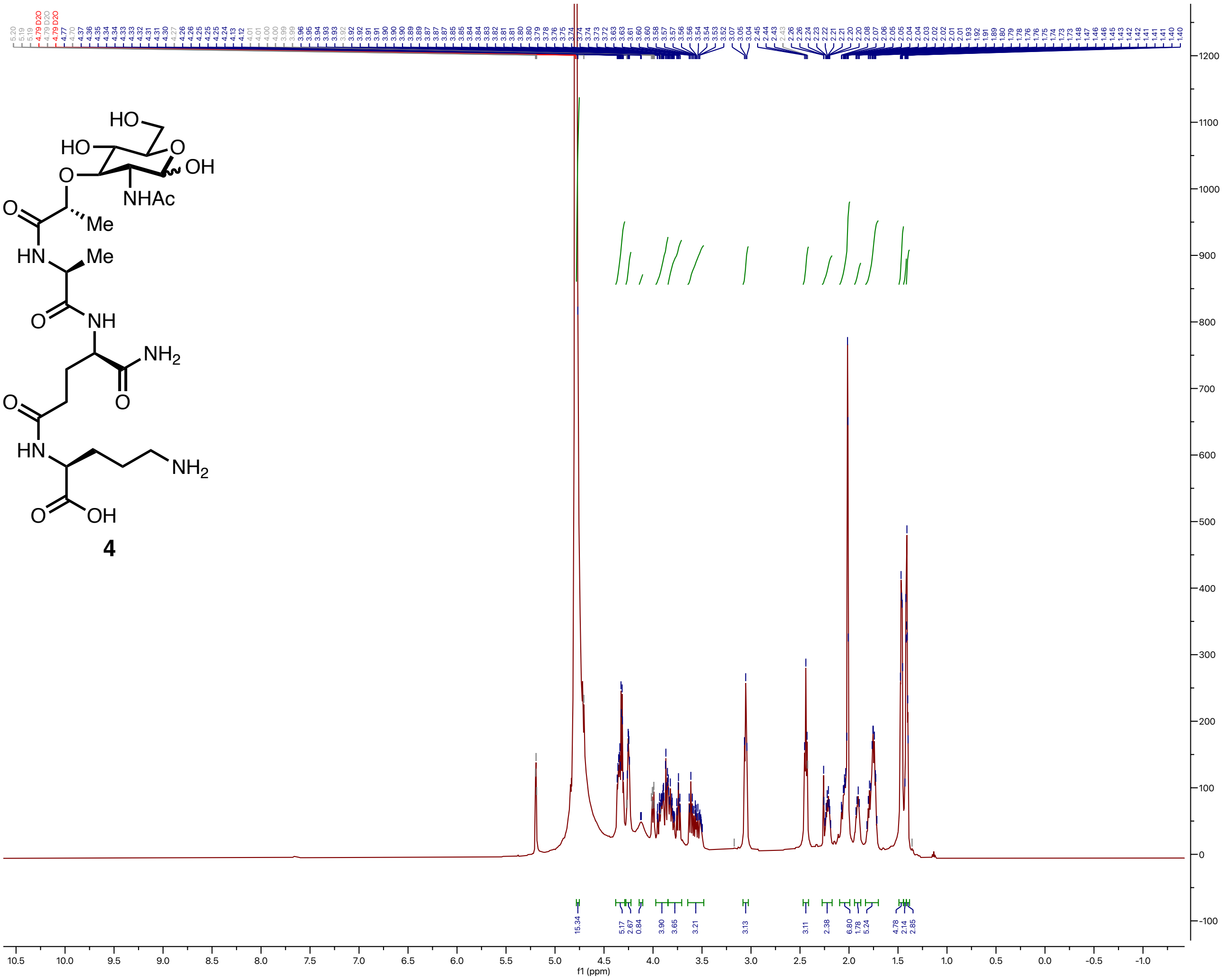

4

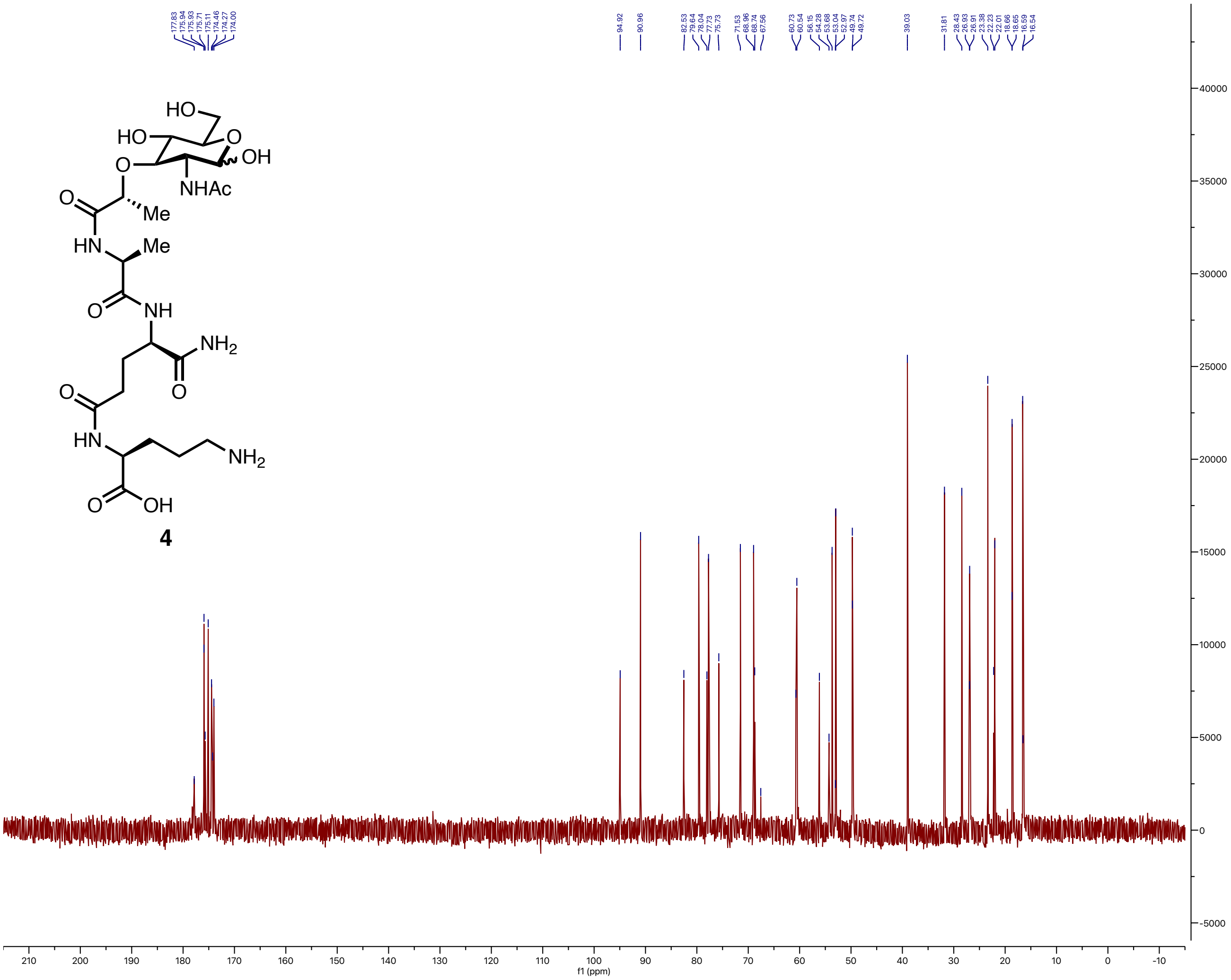

4

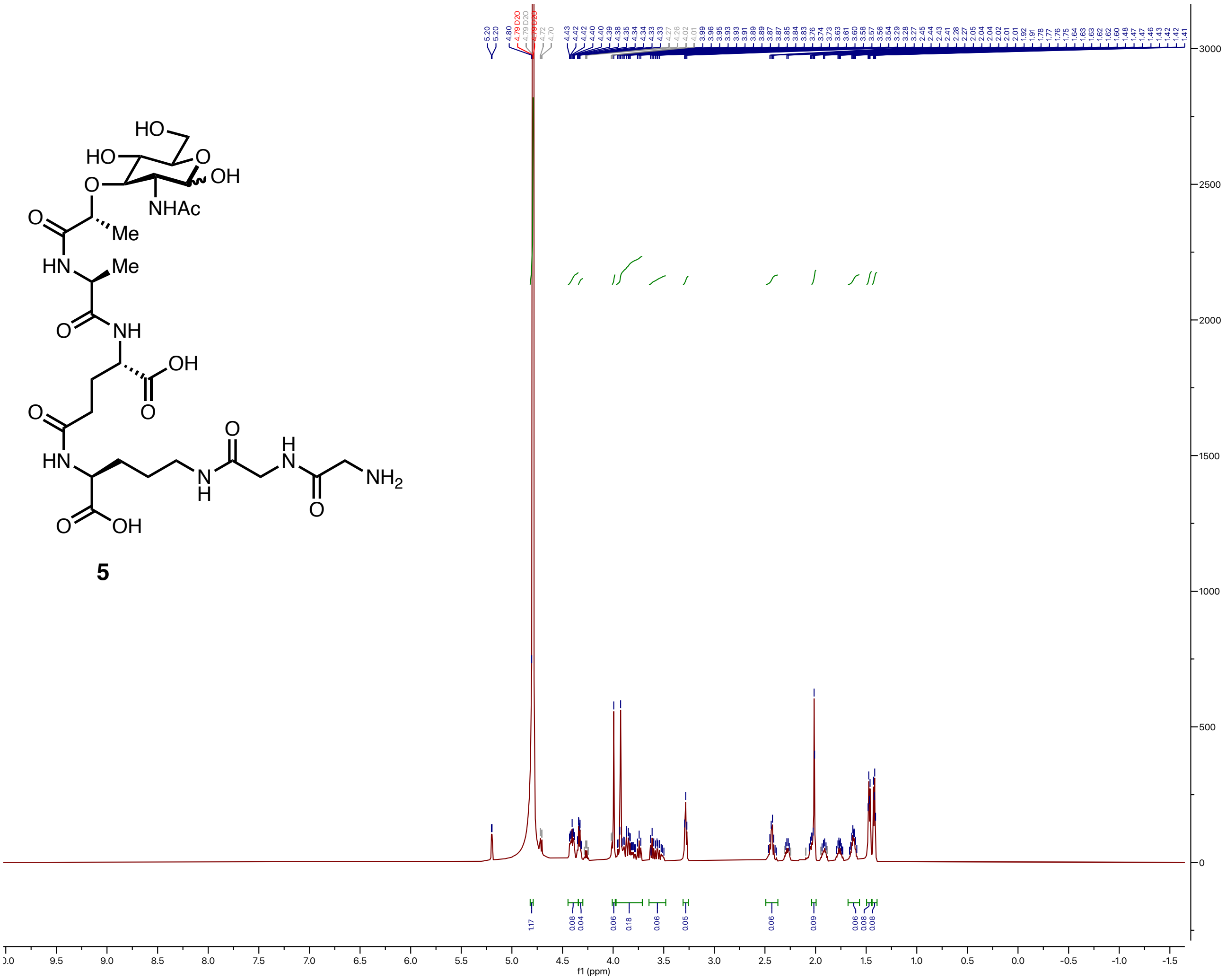

5

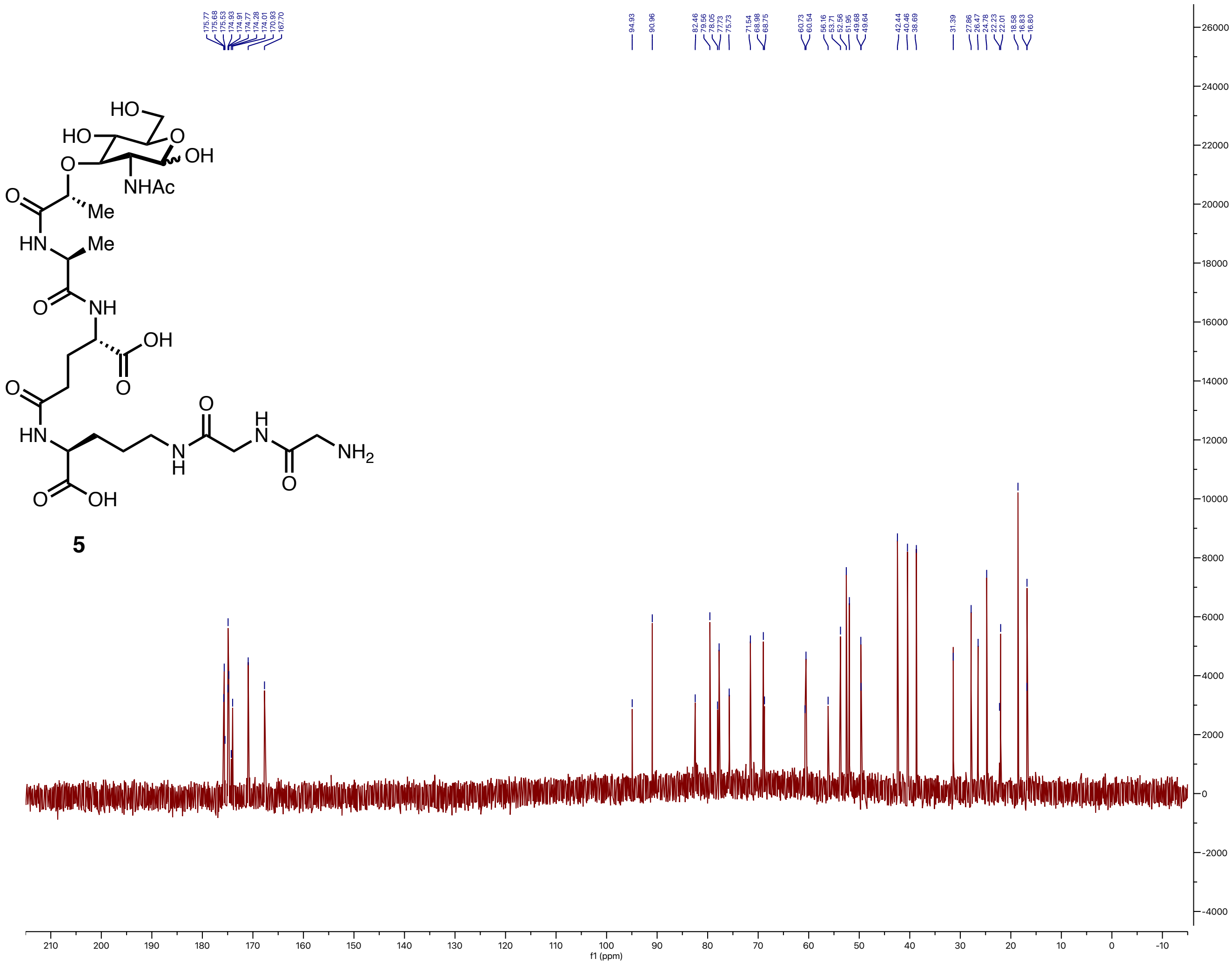

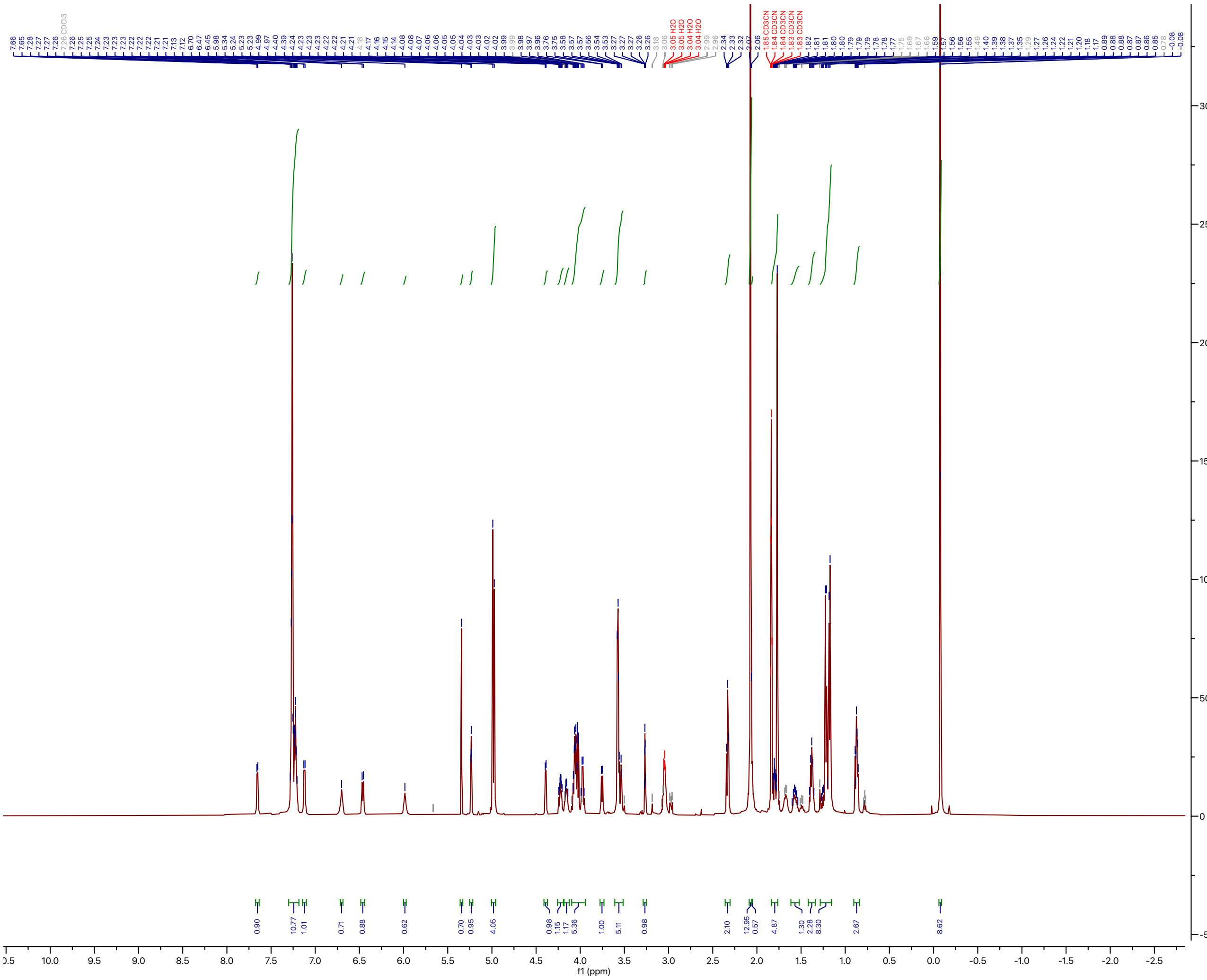

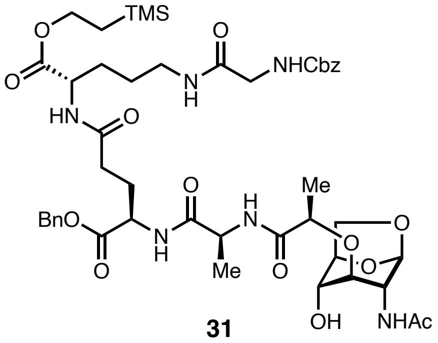

31

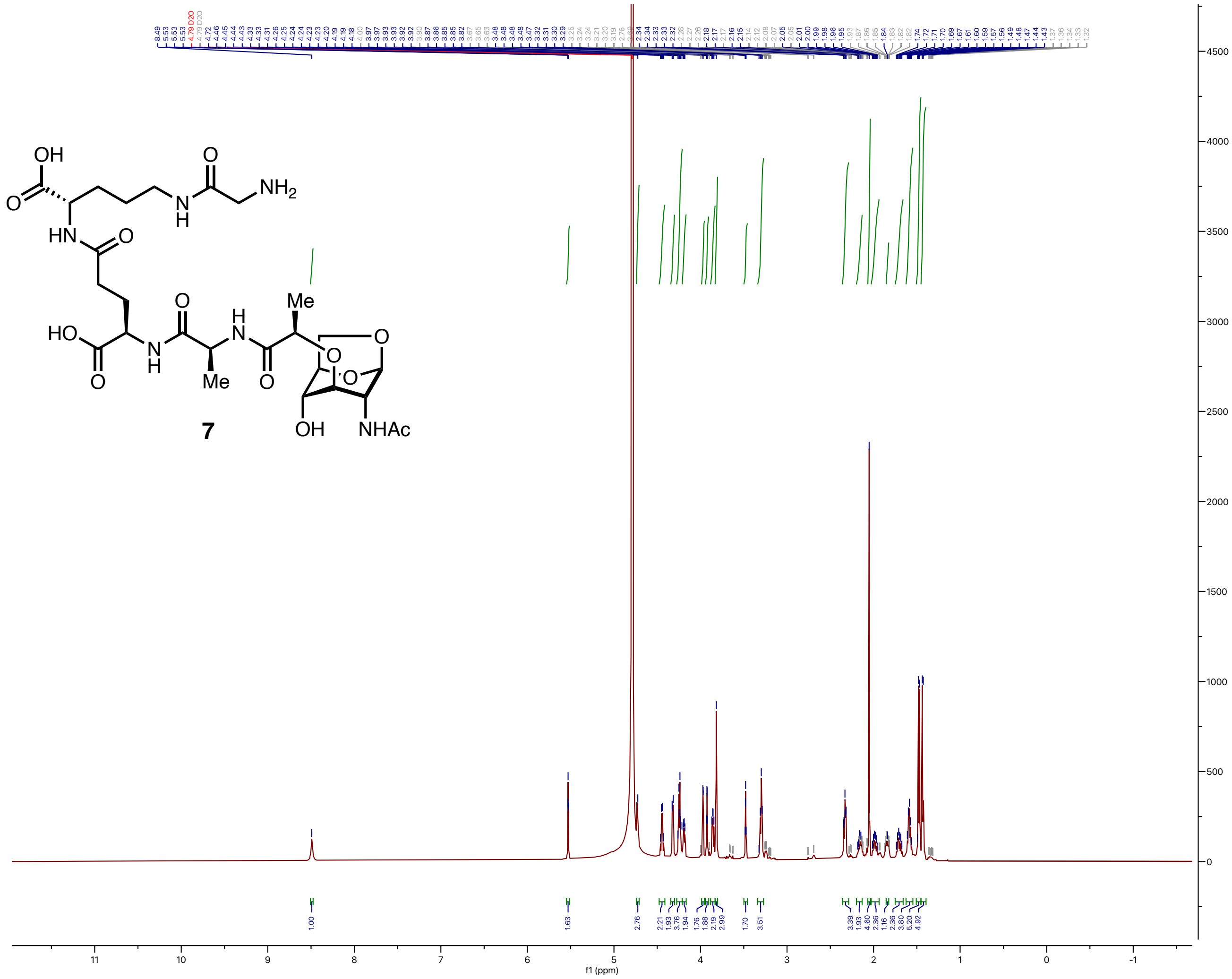
7
OH
NH2
HN
HO
Me
NHAc
f1 (ppm)

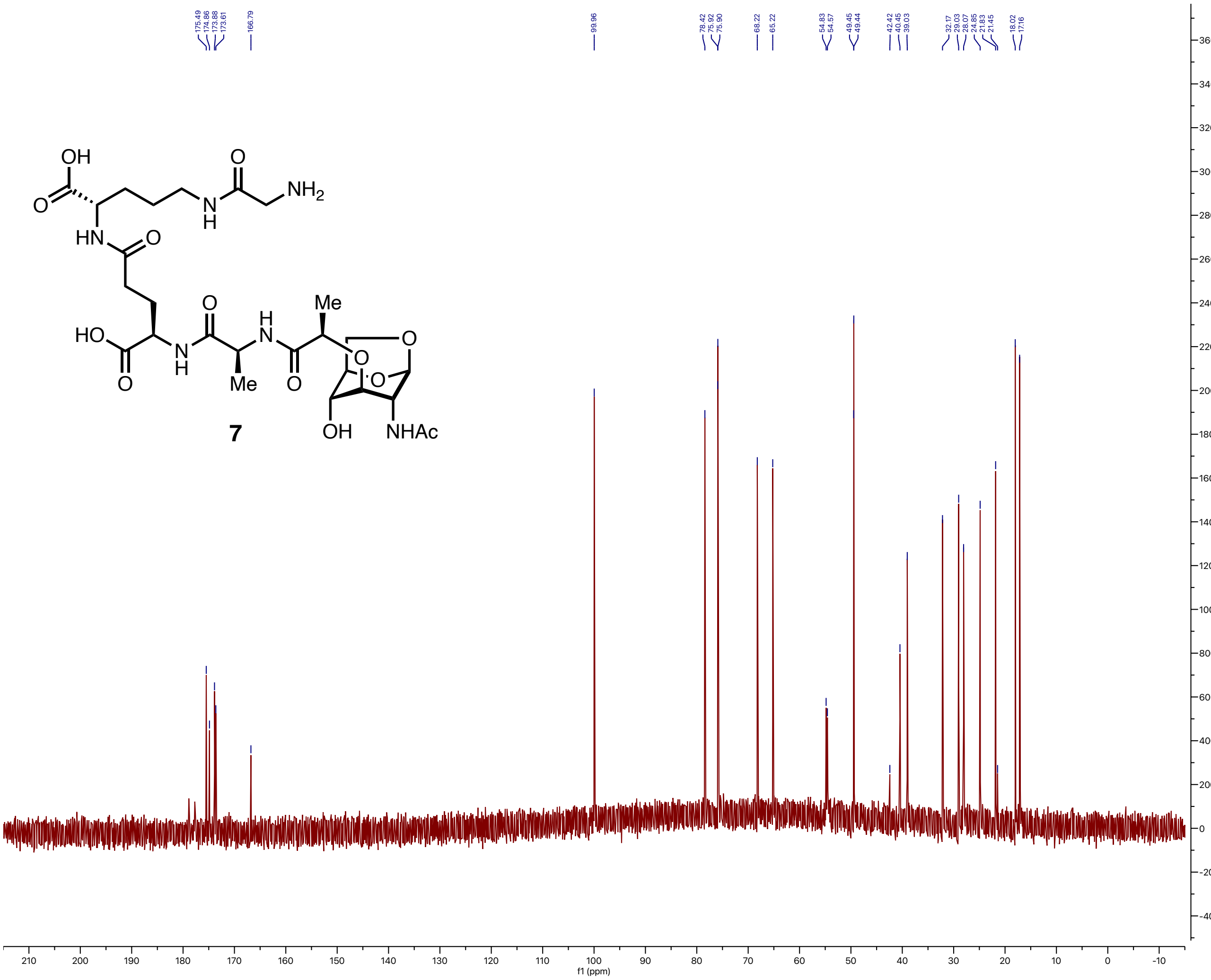
175.49
174.86
173.88
173.61
166.79
99.96
78.42
75.92
75.90
68.22
65.22
54.83
54.57
49.45
49.44
42.42
40.45
39.03
32.17
29.03
28.07
24.85
21.83
21.45
18.02
17.16
7
f1 (ppm)

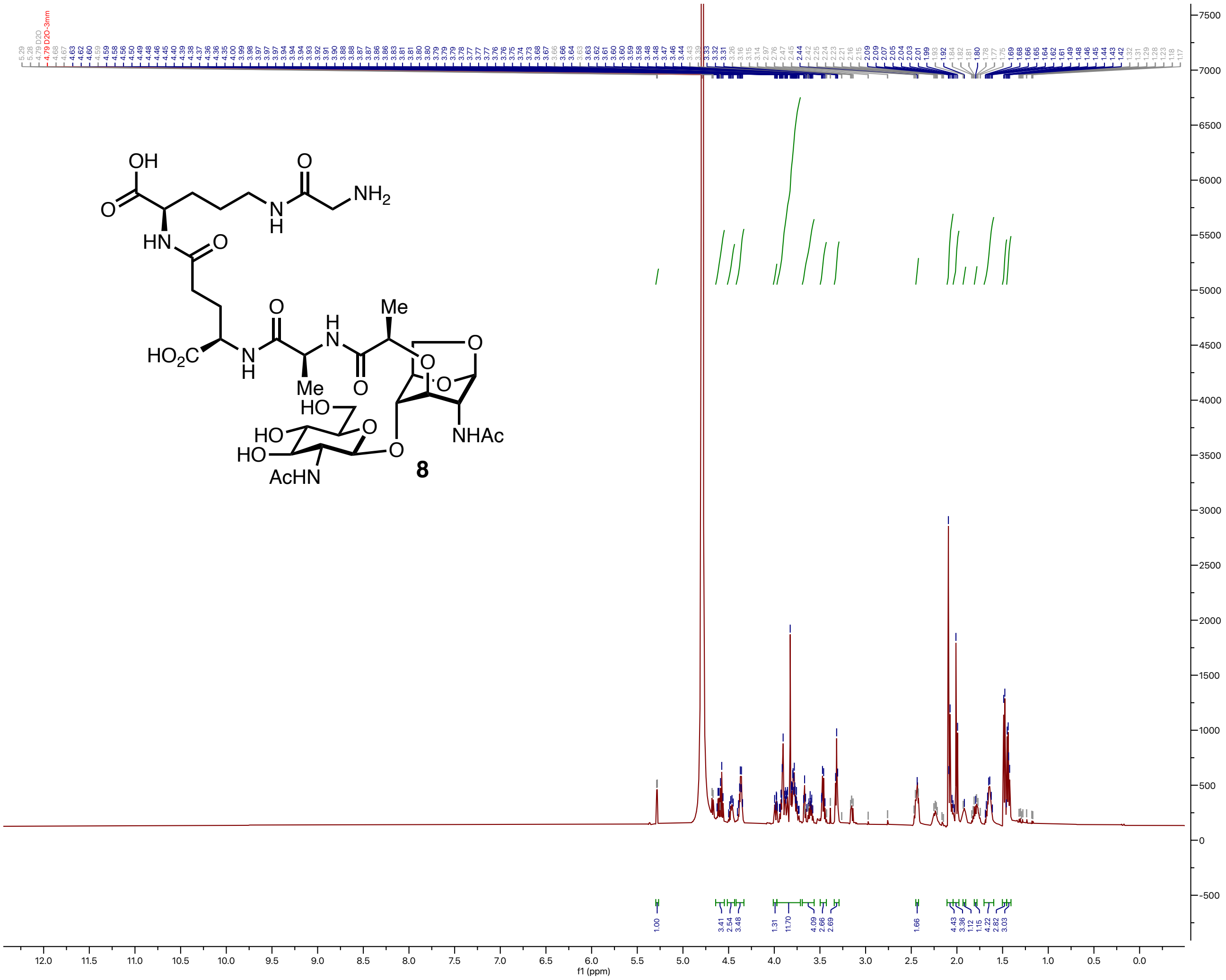

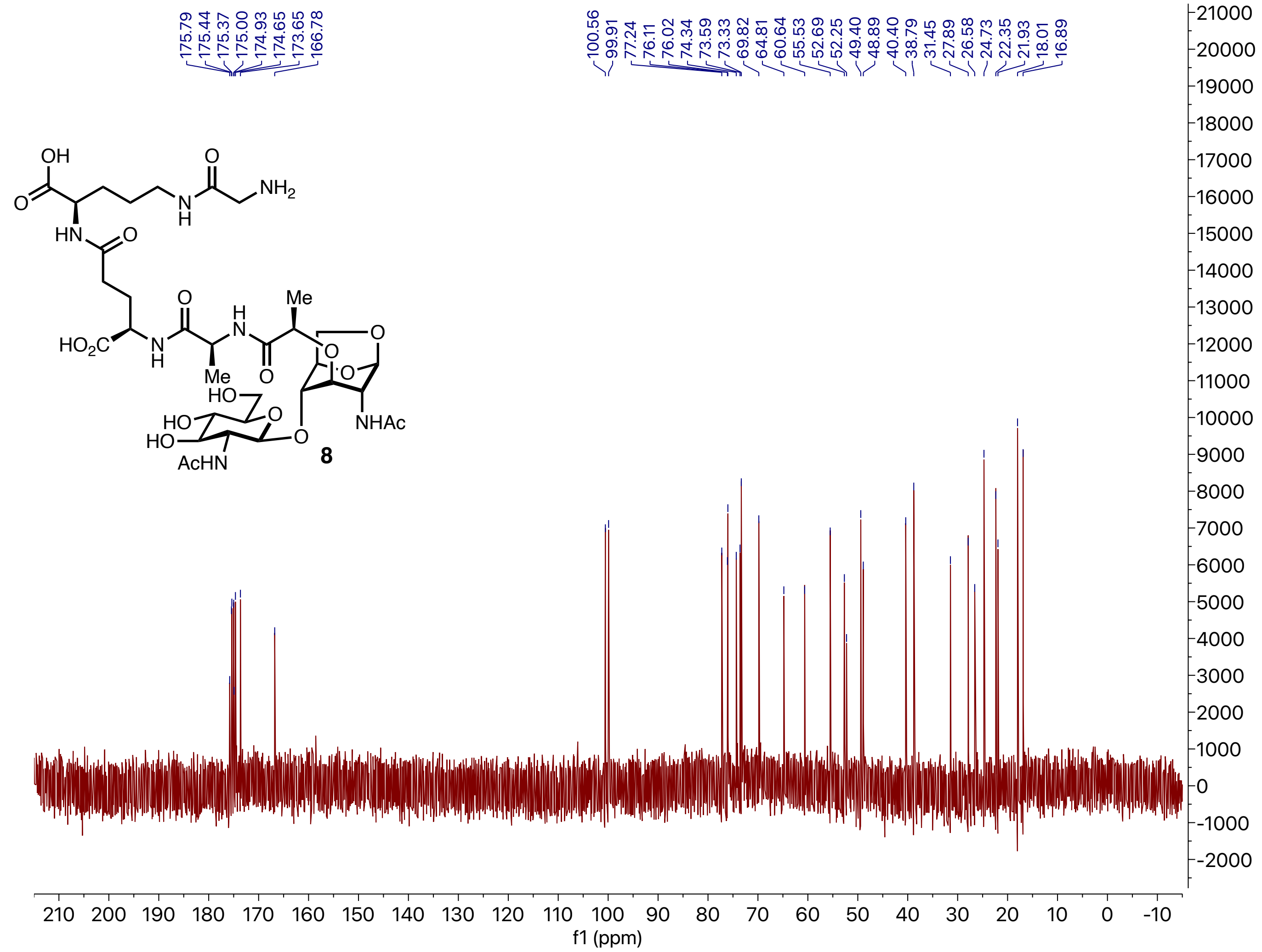
175.79
175.44
175.37
175.00
174.93
174.65
173.65
166.78
100.56
99.91
77.24
76.11
76.02
74.34
73.59
73.33
69.82
64.81
60.64
55.53
52.69
52.25
49.40
48.89
40.40
38.79
31.45
27.89
26.58
24.73
22.35
21.93
18.01
16.89
8
f1 (ppm)

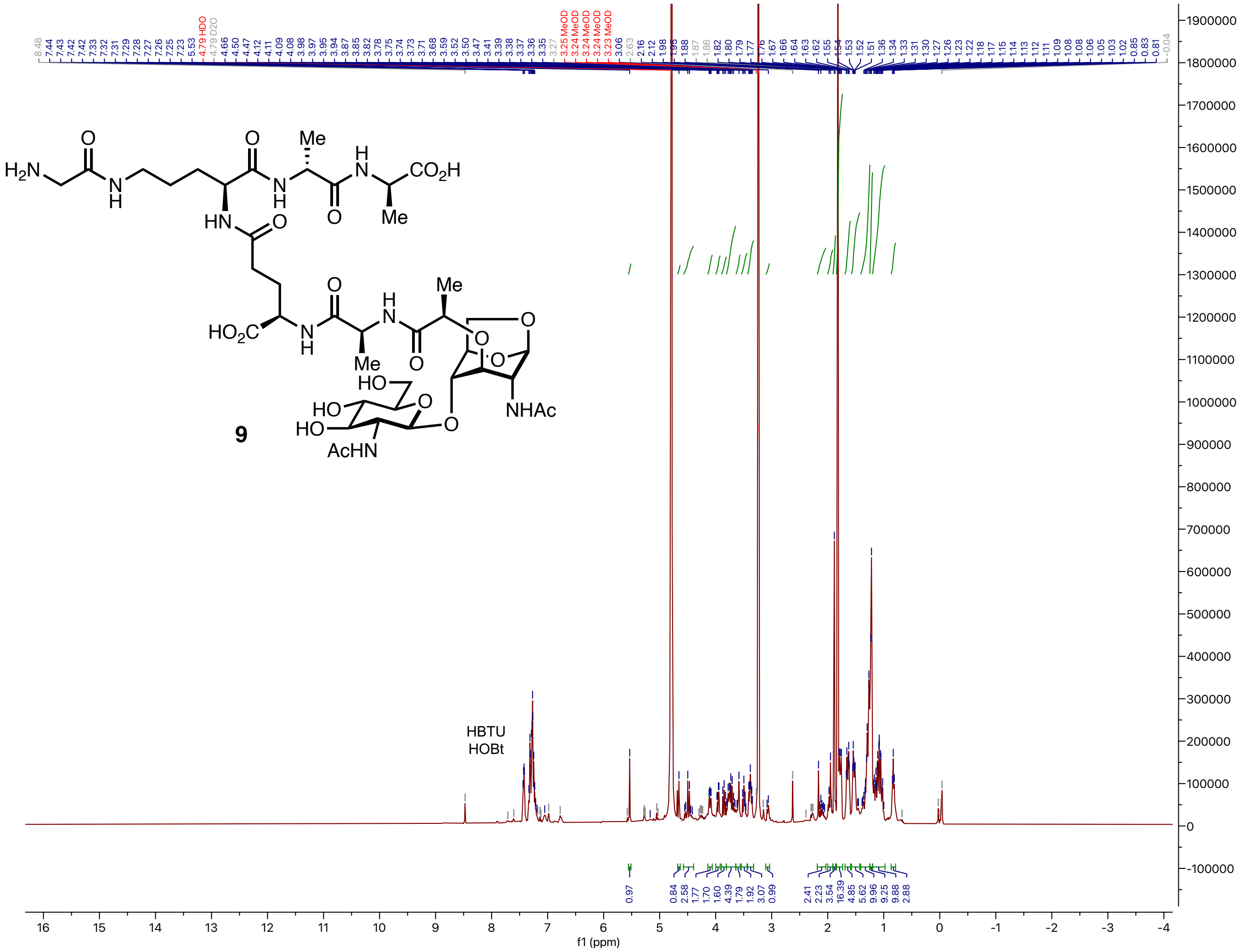
HBTU
HOBt
9
f1 (ppm)

References

1. D'Ambrosio, E. A.; Bersch, K. L.; Lauro, M. L.; Grimes, C. L., Differential Peptidoglycan Recognition Assay Using Varied Surface Presentations. *J Am Chem Soc* **2020,** *142* (25), 10926-10930.
2. Bersch, K. L.; DeMeester, K. E.; Zagani, R.; Chen, S.; Wodzanowski, K. A.; Liu, S.; Mashayekh, S.; Reinecker, H.-C.; Grimes, C. L., Bacterial Peptidoglycan Fragments Differentially Regulate Innate Immune Signaling. *ACS Central Science* **2021,** *7* (4), 688-696.
3. Chen, S.; Putnik, R.; Li, X.; Liu, S.; Zhou, J.; Guo, L.; Xu, L.; Temme, S.; Bersch, K.; Gildersleeve, J. C.; Leimkuhler Grimes, C.; Reinecker, H.-C., PGLYRP-1: Intracellular Receptor for GMTP that Controls Innate Immunity and Mucosal Recovery. Available at SSRN: https://ssrn.com/abstract=4663948 or http://dx.doi.org/10.2139/ssrn.4663948. *Immunity, Sneak Peak* **2023**.
4. Lioux, T.; Busson, R. H. C.; Rozenski, J.; Nguyen-Distèche, M.; Frère, J. M.; Herdewijn, P., Synthesis of Peptidoglycan Units with UDP at the Anomeric Position. *Collection of Czechoslovak Chemical Communications* **2005,** *70*, 1615-1641.
